# Developing theory and evidence based intervention content using the Behaviour Change Wheel, Theoretical Domains Framework, and the Person-Based Approach: A worked example for an intervention targeting sedentary behaviour with people living with obesity

**DOI:** 10.1371/journal.pone.0338196

**Published:** 2025-12-05

**Authors:** Fiona Curran, James Matthews, Gráinne O’Donoghue

**Affiliations:** School of Public Health Physiotherapy and Sports Science, University College Dublin, Dublin, Ireland; Medical Research Council, SOUTH AFRICA

## Abstract

**Introduction:**

Both sedentary behaviour and obesity are independent risk factors for poor health and quality of life. Reducing sedentary behaviour is an important public health strategy. People with moderate or severe obesity have unique challenges to reducing sedentary behaviour. The Behaviour Change Wheel (BCW) and the Person Based Approach (PBA) are complementary frameworks for intervention development aligned to these concepts. This article describes the development of content for an intervention which aims to reduce sedentary behaviour during leisure time in the domestic domain among people living with moderate or severe obesity.

**Methods:**

The BCW and PBA guided the development of the intervention content, using in-depth qualitative data with people living with obesity (n = 21). A detailed behavioural analysis conducted using the Capability, Opportunity and Motivation Behaviour (COM-B) model and Theoretical Domains Framework (TDF) provided the data for selecting appropriate content for the target user group. Content was selected using the Behaviour Change Techniques Taxonomy (BCTTv1), Theory and Techniques Tool, two systematic reviews, and key scientific literature.

**Results:**

Twenty two behaviour change techniques with potential to change SB in the domestic domain were identified, which can be delivered remotely via an online intervention. Intervention functions consist of education, persuasion, training and enablement. Strategies and guiding principles are outlined. A SB example for all 93 BCT’s in the BCTv1 is provided alongside a longlist of 50 more relevant BCTs to serve as a reference guide for future designers.

**Conclusion:**

A theory, evidence and person-based approach to develop an intervention to reduce sedentary behaviour in the domestic domain for people living with obesity is described. The BCW guide, COM-B model, TDF and BCT-Taxonomy provided the framework for selection of theoretical content. To ensure relevance for the target population, the content and mode of delivery was developed in collaboration with people with moderate to severe obesity using the PBA.

## 1. Introduction/background

Sedentary behaviour (SB) defined as “any waking behaviour characterized by an energy expenditure ≤1.5 metabolic equivalents (METs), while in a sitting, reclining or lying posture” [1], is an independent risk factor for cardiovascular disease, type 2 diabetes, obesity, cancer, and mortality [2,3]. Moreover, high levels of SB are adversely associated with body composition [4], physical activity (PA) levels, function, disability [5], health-related quality of life [5,6], cognitive function [5,7], depression [5,7,8] and anxiety [8–10].

While SB is associated with the risk of developing obesity [11], research indicates that adults who have developed obesity spend more time sedentary than adults with a healthier weight, (>1hour) [12], and time spent sedentary may also be associated with obesity severity [13]. Moreover, in adults with severe obesity (defined in study as body mass index (BMI) >35 kg/m2) one sedentary hour per day accumulated in bouts of ≥10 minutes is associated with a 15% higher odds of diabetes, 12% of metabolic syndrome and 14% of elevated blood pressure, highlighting sedentary time as a distinct health risk in this population [14]. Minimising SB is recommended nationally and internationally [3,15] with 24-hour movement guidelines promoting a healthy balance of PA, sedentary time, and sleep [16].

Interventions which specifically and solely target sedentary behaviour in their design and implementation are more effective at reducing the behaviour [17]. However, SB literature has expanded exponentially, many interventions reporting change in SB do not design interventions to target SB, but target other behaviours, e.g., physical activity [18–20] or report outcomes not consistent with the definition of SB, e.g., report change in sedentary lifestyle. There are very few published intervention studies targeting a reduction of SB outside the workplace [21] or specifically targeting people with overweight [22] or obesity [23] and none specifically targeting moderate to severe obesity, or targeting SB in the domestic domain. Hence, there is promising but limited evidence for the efficacy of SB interventions and thereby, few promising intervention components [19,21,24].

People with moderate or severe obesity have unique challenges to reducing sedentary behaviour [25]. For individuals with severe obesity, significant physical, psychological, environmental, and policy-related challenges exist beyond the home environment. Addressing these barriers requires broader systemic change to create the opportunities and support needed for reducing sedentary behaviour in this population [25]. Therefore, the domestic environment, therefore, presents an opportunity to begin to address SB for this population.

The Medical Research Council (MRC) recommend systematic use of theory and evidence for developing and implementing complex interventions [26]. Interventions are considered complex if they involve multiple interacting components, require new behaviours by those delivering or receiving the intervention, or have a variety of outcomes [27,28]. Embedding theory in intervention design and implementation, enhances identification of the effective components of interventions, which in turn enhances the accumulation of evidence [29]. However, with few exceptions to date [30,31], theoretical constructs are rarely reported or embedded in the design and delivery of SB interventions [19,24]. Although frameworks exist to guide the selection and use of theory [32,33] the development of theory-based interventions is often poorly articulated [33]. The process of identifying potential theoretical constructs, regarding the particular population and context requires considerable resources and ideally behaviour change expertise.

The Behaviour Change Wheel (BCW) developed from 19 frameworks of behaviour change, provides a comprehensive guide to identify theoretically informed intervention content and modes of delivery for behaviour change [32]. Development begins with a rigorous behavioural analysis of the target population, using the Capability, Opportunity, Motivation–Behaviour (COM-B) model and/or the Theoretical Domains Framework (TDF) [34], which contains 14 domains, the constructs of which represent the mechanisms of action of potential interventions. These constructs then map to nine intervention functions (means by which an intervention can change behaviour), seven policy categories, and 93 behaviour change techniques (BCTs) (i.e., the effective components of interventions) [35], generating intervention options and content which are likely to be more efficacious [33]. This approach has been used to develop an effective intervention to reduce SB in the workplace [30,36], and to identify the barriers and facilitators to minimising SB, for adults with moderate or severe obesity, across all domains of living (domestic, community, transportation and occupation) [25] but have not yet been systematically applied to intervention design.

While the BCW is inherently person-centred, the person-based approach (PBA) offers a complementary acceptability-focused framework that provides a rigorous process for inclusion of user needs and perceptions, to develop interventions that are feasible, acceptable and salient while avoiding impractical or intrusive elements [37,38]. Aligned to the MRC guidance to consider context and perspectives of stakeholders at each stage of the research process [25,39], the planning phase of the PBA includes two key elements; firstly, in-depth qualitative research with a purposively diverse sample of the target population and secondly identification of guiding principles. The purpose of the qualitative research, is to identify design features of an intervention, while guiding principles articulate the behaviourally contextualised design objectives and salient features of an intervention, providing a succinct summary of characteristics to support developers throughout the entire development and implementation process. Thus, the PBA is used alongside behavioural theory and analysis [36,40], aligned with best practice guidelines [26] and the detailed theoretical model planning of the BCW, is supported by a concise and succinct reference guide. This approach has been previously used to develop interventions targeting obesity management [41,42], physical activity and diet in pregnancy [43], and health behaviour change in cancer survivors [39], but yet none targeting SB have been reported.

Thus, this study combines these two complementary intervention approaches (behaviour change wheel and person-based approach) to develop theoretically underpinned intervention content and potential modes of content delivery for reducing SB in the domestic domain for people with obesity. This paper illustrates the process and provides a toolkit for theory, evidence and person based SB intervention development for use by individuals, clinicians, researchers, intervention designers and implementation scientists.

### Aim

The aim of this study is to describe a worked example of the content and potential modes of delivery of an individual level intervention targeting reduction of leisure time sedentary behaviour in the domestic domain, for people living with moderate or severe obesity.

## 2. Methods

### 2.1 Background to the study

**Fig 1:**
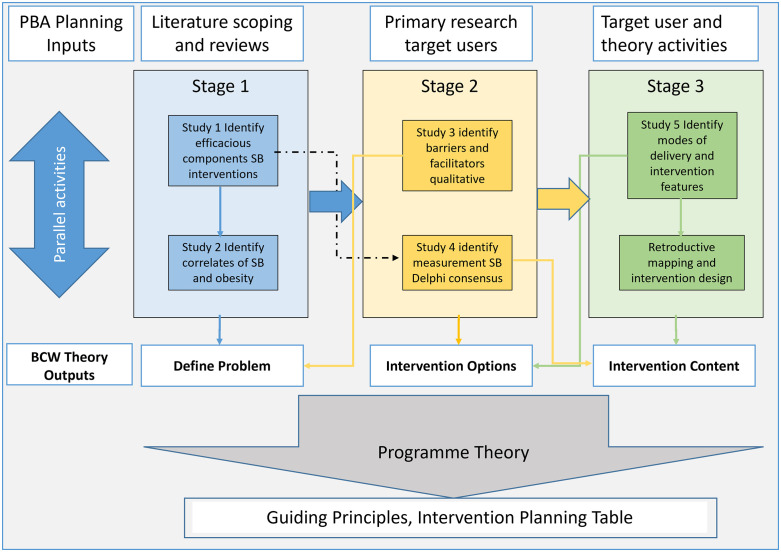
Intervention development process and contributory workstreams. Abbreviations: PBA = Person-Based Approach; SLR 1 = Systematic review of SB interventions; SLR 2 = Systematic review of correlates of SB; Qual 1 = primary qualitative study (barriers & facilitators). Qual 2 = secondary analysis of Qual 1 data; BCW = Behaviour Change Wheel.

Insights from three iterative work streams as illustrated in Fig 1, fed into the development of the intervention content. Each workstream is underpinned by the BCW and PBA methodology. Full details of workstreams one and two are available in the published studies [13,24,25,44] and are briefly outlined below. This paper reports work stream three, which comprises a synthesis and triangulation of evidence from work stream one and two, application of the three stages (8 steps) of the behaviour change wheel (described below) to identify, map and theoretically underpin a sample intervention and a secondary analysis of qualitative data gathered in workstream 2 to generate, salient design features and guiding principles (PBA).

The study received ethical approval from the University College Dublin (UCD) Human Research Ethics Committee (HREC‐LS‐21‐100). Recruitment for the qualitative study took place from 1^st^ May- 21^st^ June 2022 and all participants provided written consent online. The core research team for the intervention development consisted of FC, JM, and GO’D, while a wider project steering group reviewed and contributed to the various workstreams described below. As reflexive researchers, the all aspects of data collection, analysis and reporting were influenced the autobiographies and expertise of the research team. The research was conducted in fulfilment of a PhD for the primary researcher, FC, an experienced physiotherapist, educator and researcher, including for people with chronic and complex needs. GO’D and JM, both experienced researchers and academics with expertise in physical activity, sedentary behaviour, obesity and behaviour change, supervised the research, providing iterative, reflexive critique throughout and collectively the team generated observations and insights.

#### 2.1.1 Work stream one involved literature scoping and two separate systematic reviews.

As there were several reviews of workplace interventions to reduce SB already published [45,46], the first review focused on interventions that specifically targeted SB in adults, outside of the workplace [24]. Since evidence for SB interventions in adults with obesity was also lacking, a second review to identify the potential correlates and determinants of SB in adults living with obesity [13] was conducted to inform the future qualitative work streams and identify potentially modifiable associations.

#### 2.1.2 Work stream two involved primary research with target users and stakeholders, and comprised two studies, full details of which are available in the separately published papers.

Briefly, the first study, a consensus study, engaged a panel of SB experts, each with multiple relevant publications in peer reviewed journals, and wider global SB researchers from academia and clinical practice, in a Delphi process to identify and validate a core outcome set (COS) to report (what, how, when to measure) in SB interventional studies (CROSBI consensus study) [44]. This study was conducted in response to the findings from the first systematic review which showed that heterogeneity of descriptors and outcomes measured and reported in SB research hinder the meta-analysis of data and accumulation of evidence [24].

The second, a qualitative study involved in-depth, semi-structured interviews with adults with moderate or severe obesity (n = 21) to explore barriers and facilitators to interrupting or minimising SB across all domains of living and map them to the COM-B and TDF [25]. This primary study, which was grounded in a critical realism and reflexive thematic analysis, provided the rich contextual foundation for understanding the behaviour in context with the target population.

#### 2.1.3 Work stream three reported here, involved further target user research and theory based activities including synthesis and triangulation of evidence gathered in work streams one and two, and mapping of contextualised data to theoretical intervention content using the three stage, eight step methodology of the behaviour change wheel, the theory.

A secondary analysis of qualitative data gathered in work stream two was conducted to inform the potential mode of delivery and features of the intervention (final step of BCW). Finally, guiding principles and an intervention planning table were developed (PBA). Throughout the intervention mapping and planning, each member of the research team reviewed the evidence independently, and together engaged in an iterative process to discuss and agree the theoretical content and features appropriate to the context.

**Fig 2:**
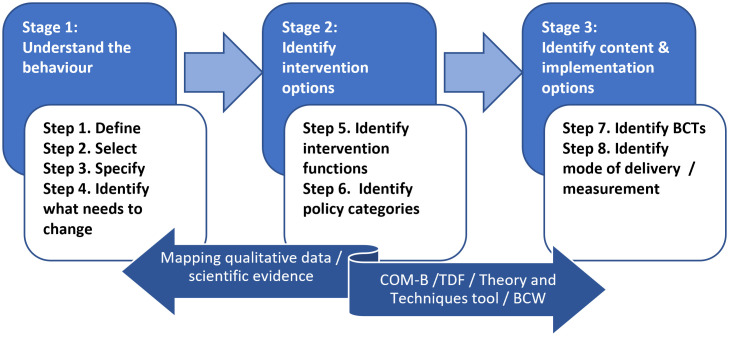
Stages and steps of The Behaviour Change Wheel with mapping methodology. Abbreviation: BCTs = Behaviour Change Techniques; COM-B = Capability Opportunity, Motivation-Behaviour (model); TDF = Theoretical Domains Framework.

***Developing intervention content using the Behaviour Change Wheel.*** To develop an evidence and theory based intervention targeting SB reduction in the population of adults with obesity we followed the three stage process of the behaviour change wheel [44,47]. These stages are 1) Understand the behaviour 2) Identify intervention options and 3) Identify content and implementation options. Each phase includes multiple steps, eight in total, as identified in Fig 2.

*Stage one; understand the behaviour (steps 1-4a).* To understand the behaviour and identify what individual, social, environmental or policy factors need to change to reduce SB in the target population (people with obesity), a detailed behavioural analysis was conducted, informed by the aforementioned systematic reviews [13,17,24] and other seminal research [17–19,21,48] and in-depth reflexive thematic analysis of semi structured qualitative interviews with 21 people with obesity. Barriers and facilitators to changing SB across all domains of living previously published [25] were refined to focus upon those that applied particularly to the domestic domain and mapped to both the COM-B and TDF.

*Stage two; identify intervention options (steps 5–6).* Outputs generated from stage 1 (steps 1-4a) form the basis for selection of potential intervention options (intervention functions and support policies). An intervention function is a broad category of ‘means by which an intervention can change behaviour’ [47]. The BCW contains nine intervention functions (education, persuasion, incentivisation, coercion, training, enablement, modelling, environmental restructuring and restrictions), which map to both the COM-B and the TDF domains. FC initially mapped the COM-B and TDF constructs to the intervention functions in the BCW. Subsequently the research team independently reviewed the mapping and evidence, then agreed the inclusion of intervention functions based on discussion of the contextual behavioural analysis, scientific evidence and the APEASE criteria [47].

Similarly, of the seven policy categories identified in the BCW, potential candidate policy categories were mapped to the intervention functions by FC and considered in context by the research team using the APEASE (acceptability, practicability, effectiveness, affordability, side-effects, equity) criteria [47]. Categories were agreed once consensus was reached.

*Stage 3; Identify content and implementation options (mode of delivery) (steps 7–8).* Consideration of all BCTs is recommended for development of any intervention [47]. Therefore an example relevant to SB change was generated for each of the 93 BCTs listed in, the behaviour change technique taxonomy V1 (BCTT v1) [31]. (See Table S7 in S1 File).

Subsequently, BCTs with most relevance and potential were identified using a number of methods. Firstly, BCTs were identified by mapping the six candidate intervention functions to the most frequently used BCTs listed in the BCW. Secondly, BCTs were identified by direct mapping to the TDF domains from the qualitative analyses, using the expert consensus from the BCW and also using the behaviour change theory and techniques tool [49]. BCTs were also identified from the SLRs and key scientific literature. Finally, further discussion and consideration of less frequently used BCT to identify any that were ‘missing’. Results were tabulated using MS Excel, cross-referenced and iteratively discussed among the research team, to develop a longlist of potentially effective BCTs. (See Table S8 in S1 File). Further triangulation of evidence and iterative discussion among the research team to was used to refine a shortlist of BCTs relevant to the targeted intervention functions. Measurement of SB outcomes was informed by a stakeholder consensus study conducted in workstream 2, (full details in the published paper) [47].

Finally, to identify potential modes of delivery and intervention features, a secondary analysis of the primary data collected in workstream 2 was conducted and is reported here.

*Secondary analysis of primary qualitative data.* The original qualitative interviews, which were conducted with a purposively diverse range of adults living with moderate or severe obesity, (n = 21) included 15 females and six males, with ages ranging from 31 to 64 years, and BMI ≥ 35 kg/m2, further demographics are detailed elsewhere [25]. To explore intervention suggestions, modes of delivery and intervention features, two specific open questions regarding potential interventions, were included at interview, with reflexive follow up questions by the interviewer (FC); (If someone was trying to help you to reduce your sitting/sedentary time, are there any things that would be useful/ off-putting? What worked/ would work for you to reduce SB?). These questions are aligned to the PBA specifically to identify salient intervention features that are acceptable or unacceptable from a user perspective. These questions were not specific to the domestic domain since the original qualitative study explored all domains of living. The original verbatim transcripts were re-analysed and coded for intervention content, features, modes of delivery and guiding principles. The research team then mapped the theoretical content to modes of delivery and intervention features to design a sample intervention.

## 3. Results

### 3.1. Stage 1 understand the behaviour

*Problem statement;* Adults who have developed obesity spend more time sedentary than adults with a healthier weight, (>1hour) [12], and in adults with severe obesity, one sedentary hour per day accumulated in bouts of ≥10 minutes is associated with a 15% higher odds of diabetes, 12% of metabolic syndrome and 14% of elevated blood pressure, highlighting sedentary time as a distinct health risk in this population, who accumulated a median 6.5 hours per day sedentary time in bouts ≥10 minutes [14]. The increased levels of SB, alongside the low levels of physical activity (PA) in this population, is concerning [5,50]. International PA guidelines now include advice to minimise SB, and more recently, 24-h movement guidelines promote a healthy balance of PA, sedentary time, and sleep [15,16]. Furthermore, interventions directly targeting SB have greater potential to change SB than interventions targeting combinations of SB and PA or SB and other lifestyle interventions [19,47].

#### Step 1. Define the problem in behavioural terms.

To define the problem in behavioural terms we identified the target population (people living with moderate or severe obesity) and the behaviour itself (sedentary behaviour), reviewed the scientific literature, completed two systematic reviews [13,24] (work stream 1) and a qualitative study [25] (work stream 2). Work stream one identified potential association of SB with severity of obesity and number of co-morbidities [13] but a lack of evidence regarding patterns of SB for people with obesity, a lack of evidence for non-workplace SB interventions and inconsistent measurement of SB outcomes [24]. Work stream two identified how best to measure outcomes [44], barriers to SB change in all domains of the TDF and opportunity for SB change in all domains of living (occupational, domestic, transportation and community) for people with obesity [25]. (See Table S1 in S1 File).

#### Step 2. Select the target behaviour.

Following review of the literature and qualitative evidence, we identified a ‘long list’ of candidate behaviours which could influence SB in people with obesity in various contexts and domains of living. Selecting the target behaviour was informed by the qualitative interviews (workstream 2). The list was systematically reduced by iterative discussion among the research team, to a set of core behaviours which were pragmatic and achievable as recommended in the BCW [47]. Promising behaviours were shortlisted by consideration of likelihood of change being achieved, likely impact of change, feasibility and acceptability to people with obesity, and ease of measurement. Potential candidate behaviours included physical activity, which was out-ruled by the problem statement, and sleep behaviour, which was out-ruled as not likely to influence SB for all of the target population as a first step in behaviour change. Postural and energy components of SB change were selected, meaning that the target behaviour chosen is to exchange SB for increased seated energy expenditure or standing, preferably with movement. (See Table S2 in S1 File).

#### Step 3. Specify the target behaviour.

Once the target behaviour was selected and defined as ‘standing up from sitting/ reclining, or taking movement breaks’, a more detailed description regarding context and dependencies was defined. Namely, who performs the target behaviour, what do they need to do differently, and when, where, how often, and with whom will they do it (Table 1).

**Table 1:** Specify the target behaviour.

Describe the target behaviour according to who needs to do what, when, where, how often and with whom	
**Target behaviour**	Non-SB at home (domestic domain)
	Stand up from sitting/ Add movement break (encourage upright but include seated activity)
***Who* needs to perform the behaviour?**	People with obesity
***What* do they need to do differently to achieve the desired change?**	Break-up/ reduce low energy prolonged SB
	(sitting/ reclining/ lying time while awake)
***When* do they need to do it?**	Every day, (at least hourly, morning, afternoon, evening).
	During leisure time, e.g., watching TV, using screens for leisure, reading, voluntary activities that include computer use, online or administrative home tasks, socialising at home.
***Where* do they need to do it?**	In or around the home
***How often* do they need to do it?**	Every 30–60 minutes while awake
***With whom* do they need to do it?**	Alone, behaviour is not dependent on others

#### Step 4: identify what needs to change.

To identify what individual, social, environmental or policy factors need to change to reduce SB in the target population (people with obesity), barriers and facilitators to changing SB previously published [25] were refined to focus upon those that applied particularly to the domestic domain, by reviewing the original transcripts and analyses and mapping to both the COM-B and TDF. Barriers and facilitators to reducing sedentary behaviour during leisure time, were identified in all 14 domains of the TDF and all six components of the COM-B, the relevance of which is identified. (See Tables S3 and S4 in S1 File).

Therefore, physical and psychological capability, physical and social opportunity, and reflective and automatic motivation need to change in order for the target behaviour ‘standing up from sitting/ reclining’ or ‘take movement breaks’ to occur. Participants, for example, believed that they lacked the physical capability to reduce SB due to pain, physical or medical co-morbidities, or because of the effort involved in moving a larger body. They believed that negative emotion (e.g., fear of movement, feeling stressed), and poor psychological wellbeing reduced motivation to move, and internalised weight bias appeared to affect motivation whereby little value was attributed to movement without weight loss (e.g., breaking sedentary bouts with comfort breaks or domestic tasks). Furthermore, SB was conflated with physical inactivity, and most participants believed that non SB was synonymous with physical activity. Even when participants perceived value in non SB, some lacked readiness to change, particularly in evening sedentary time which they associated with necessary rest. Most participants cited tiredness in the evening, making an effort to complete work, physical activities or domestic tasks earlier in the day. Participants believed that knowledge about health consequences of SB and acceptance of obesity as a disease could facilitate reduced SB.

### 3.2 Stage 2: identify intervention options

#### Step 5: Identify intervention functions.

Table 2 presents each TDF domain mapped to the appropriate intervention functions, alongside the relevance of each domain. The intervention functions chosen as most relevant and meeting APEASE criteria were education, persuasion, training and enablement, while the functions of environmental restructuring and modelling were also considered of relevance but a lesser priority in the domestic domain. The functions of coercion and incentivisation were deemed unacceptable or impractical in the domestic domain. (See Table S5 in S1 File).

**Table 2:** Identify intervention functions.

COM-B component	TDF Domain *definition*	Relevance of domain (what needs to change) From qualitative data/ scientific literature/ Iterative discussion	Intervention functions
Physical capability	**Physical skills** *An ability or proficiency acquired through practice*	Have the physical skills, strength or stamina to stand or move from reclining/ sitting (consider pain, mechanical and medical barriers) and/ or increase energy expenditure when seated (seated movement)	Training
Psychological capability	**Knowledge** *An awareness of the existence of something*	Know what SB/ non-SB is and how to break SB. Know complexity of factors influencing SB (obesity, mental, physical health, sleep, nutrition, PA) Know about obesity (as disease, complexity, treatments, care, set-point) Know how/ where to access supports (movement, medical, mental wellbeing) Know how to adapt exercises/ movement Know health consequences (SB, Obesity) Know health benefits (Non SB, LPA, comfort breaks, mobility, functional ability) Know how to create and apply ‘if- then’ rules to prompt non SB (standing/ movement breaks)	Education
	**Cognitive and interpersonal skills** *An ability or proficiency acquired through practice*	Be able to prioritise and advocate for personal wellbeing/ non SB in the home/ familial context (e.g., reframe comfort breaks/ tasks and chores/ caregiving)	Training
	**Memory, attention and decision processes** *The ability to retain information, focus selectively on aspects* *of the environment and choose between two or more alternatives*	Notice and remember to stand or take a movement break Believe that non SB is valuable to health independent of PA, body weight, nutritional status Be able to choose/ prioritise non SB over competing behaviours, (particularly when demotivated, tired)	Environmental restructuring Training Enablement
	**Behavioural regulation** *Anything aimed at managing or changing objectively observed or measured actions*	Develop skills of smArt goal setting, self-monitoring and action planning and applying ‘if-then’ rules (note; some PwO are anti-goals/ have negative experience of goal setting, therefore, must be achievable & plan for when not met)	Education Training Modelling Enablement
Physical opportunity	**Environmental context and resources** *Any circumstance of a person’s situation or environment that discourages or encourages the development of skills and abilities, independence, social competence, and adaptive behaviour*	Have/ use seating which is easy to stand from, and/ or equipment to increase energy expenditure when using screens (e.g., standing during screentime or seated PA equipment) Provide materials/ resources which demonstrate/ develop skills/ support for non-SB	Training Restriction Environmental restructuring Enablement
Social opportunity	**Social influences** *Those interpersonal processes that can cause individuals to change their thoughts, feelings, or behaviours*	The opportunity to engage/ practice non SB with peers PwO and HCPs skilled and informed in the complexity of obesity and the barriers to non-SB. Access to engage with empathetic peers (PwO) with shared experiences. Access to HCPs skilled and informed in the complexity of obesity and the barriers to non-SB (& timely mental health support)	Restriction Environmental restructuring Modelling Enablement
Reflective motivation	**Professional/social role and identity** *A coherent set of behaviours and displayed personal qualities of an individual in a social or work setting*	Develop identity as non-sedentary adult, (independent of past current identity as ‘physically active/ inactive’, ‘sporty’ or ‘lazy’) Be able to identify SB in competing social/ familial roles, (carryover non-SB in these roles) Be able to boundary professional/ voluntary activities at home (carryover non-SB in these roles)	Education Persuasion Modelling
	**Beliefs about capabilities** *Acceptance of the truth, reality, or validity about an ability, talent, or facility that a person can put to constructive use*	Believe that non-SB is achievable despite some limitations (physical/ medical/ pain/ body weight). Believe that consistent non-SB will require improved cognitive and self-regulation skills.	Education Persuasion Modelling Enablement
	**Optimism** *The confidence that things will happen for the best or that desired goals will be attained*	Develop confidence that non-SB is achievable and worthwhile independent of weight/ PA/ obesity Reduce unrealistic optimism of weight loss	Education Persuasion Modelling Enablement
	**Beliefs about consequences** *Acceptance of the truth, reality, or validity about outcomes of a behaviour in a given situation*	Believe that consistent non-SB will benefit physical and psychological health. Believe that SB contributes to deterioration in physical and mental health and obesity. Believe that non-SB has health benefits/ consequences independent of PA/ obesity/ nutritional intake	Education Persuasion Modelling
	**Intentions** *A conscious decision to perform a behaviour or a resolve to act in a certain way*	Develop and stabilise intention/ decision to reduce SB	Education Persuasion Incentivisation Coercion Modelling
	**Goals** *Mental representations of outcomes or end states that an individual wants to achieve*	Set realistic achievable (smArt) goals, independent of past or future/ failures	Education Persuasion Incentivisation Coercion Modelling Enablement
Automatic motivation	**Reinforcement** *Increasing the probability of a response by arranging a dependent relationship, or contingency, between the response and a given stimulus*	Reduce negative reinforcement associated with non SB especially pain (pacing vs boom bust) Reinforce non sedentary routines and habits Reinforce negative consequences of SB (physical/ emotional) (could be unethical given stigma/ bias/shame/effect on health)	Training Incentivisation Coercion Environmental restructuring
	**Emotion** *A complex reaction pattern, involving experiential, behavioural,* *and physiological elements, by which the individual attempts to deal with a personally significant matter or eve*nt	Reduce negative emotion associated with non SB at home fear of movement/ falling/ judgement/ pain/ non achievement. Promote acceptance of self/current ability body/ obesity Identify enjoyable non-SBs Encourage awareness of affect on SB and non SB Develop awareness and coping skills for negative emotions particularly internal bias	Persuasion Incentivisation Coercion Modelling Enablement

**Behavioural diagnosis:** Physical and psychological capability, physical and social opportunity, and reflective and automatic motivation need to change in order for the target behaviour ‘standing up from sitting/ reclining or take movement breaks’ to occur.

Opportunities for intervention exist in all theoretical domains.

**Intervention functions chosen:** Education; Persuasion; Training; Enablement;

+/- Environmental restructuring; Modelling;

#### Step 6: identify policy categories.

The research team used the APEASE criteria mapped from the BCW to consider the policy categories most likely to support the chosen intervention functions (education, training, persuasion, enablement) in the domestic domain. Policy categories deemed most likely to support the intervention functions were communication and marketing, development/ adoption of guidelines and service provision, while regulation, legislation, fiscal measures and environmental restructuring were considered either not practical or not acceptable. (See Table S6 in S1 File).

### 3.3 Stage 3: identify content and implementation options

#### Step 7: identify behaviour change techniques (BCTs).

Twenty two BCTs were identified by mapping the six candidate intervention functions to the most frequently used BCTs listed in the BCW. Independently, 43 BCTs were identified by direct mapping to the TDF domains from the qualitative analyses, using the expert consensus from the BCW and 38 BCTs were identified using the behaviour change theory and techniques tool [49]. Further consideration of all 93 BCTs by the research team identified four further BCTs less frequently used but deemed of relevance to this population, relating to identity and emotion. Finally, 24 BCTs were identified from the SLRs and key scientific literature.

Following tabulation, triangulation and iterative discussion, a longlist of 50 potentially effective BCTs (see Table S8 in S1 File) and a shortlist of 22 BCTs relevant to the six targeted intervention functions were agreed (Table 3).

**Table 3:** BCT’s targeting change in leisure time SB in the domestic domain for people with obesity (shortlist).

BCT No. (Source)	BCT Label	Definition	Example for SB reduction	Intervention functions targeted
**1.2** **(a,c,d,e)**	***Problem solving***	Analyse, or prompt the person to analyse, factors influencing the behaviour and generate or select strategies that include overcoming barriers and/or increasing facilitators (includes ***‘Relapse Prevention’ and ‘Coping Planning’***)	Identify specific triggers (e.g., feeling low/ anxious/tired) that initiate and sustain prolonged sedentary/ sitting time and develop strategies for avoiding environmental triggers or for managing negative emotions, such as anxiety, that motivate SB	Enablement
**1.4** **(a,b,d,e)**	***Action planning***	Prompt detailed planning of performance of the behaviour (must include at least one of context, frequency, duration and intensity). Context may be environmental (physical or social) or internal (physical, emotional or cognitive) (includes ***‘Implementation Intentions’***)	Prompt planning taking a movement breaks from evening TV, identifying ad breaks as cues to move, allowing one break to be ignored, move every second break when feeling tired/ stressed.	Enablement
**2.2** **(a,b,c,d,e)**	***Feedback on behaviour***	Monitor and provide informative or evaluative feedback on performance of the behaviour *(e.g., form, frequency, duration, intensity)*	Inform the person of the time they spent sedentary in minutes per day, and how many prolonged bouts or how many movement breaks the took.	Education Persuasion Incentivisation Coercion Training
**2.3** **(a,b,c,d,e)**	***Self-monitoring of behaviour***	Establish a method for the person to monitor and record their behaviour(s) as part of a behaviour change strategy	Ask the person to use a daily diary to record when they start and stop sitting, and how many movement breaks they take. Use a wearable device (accelerometer with postural component) with phone app showing time spent sedentary, sedentary bouts and breaks.	Education Incentivisation Coercion Training Enablement
**3.1** **(a,b,c,d,e)**	***Social support (unspecified)***	Advise on, arrange or provide social support (e.g., from friends, relatives, colleagues,‘ buddies’ or staff) or non-contingent praise or reward for performance of the behaviour. It includes encouragement and counselling, but only when it is directed at the **behaviour**	Advise the person to chat to family/ housemates when they feel like screen time, Arrange for housemate to encourage movement breaks/ non SB	Enablement
**3.2** **(a,b,c,d,e)**	***Social support (practical)***	Advise on, arrange, or provide **practical** help *(e.g., from friends, relatives, colleagues, ‘buddies’ or staff) for performance of the behaviour*	Ask the person’s housemate not to bring refreshment to the person when at screens/ when watching T.V or to ask the person to bring refreshment for both (e.g., hot beverage). Arrange for person to attend online seated movement class, e.g., chair yoga	Enablement
**4.1** **(a,c,d,e)**	***Instruction on how to perform a behaviour***	Advise or agree on how to perform the behaviour (includes ‘**Skills training**’)	Advise the person how to increase seated energy expenditure/ how often to take sedentary breaks.	Training
**5.1** **(a,b,c,d,e)**	***Information about health consequences***	Provide information (e.g., written, verbal, visual) about health consequences of performing the behaviour	Explain that non SB maintains/ improves mobility/ cardiovascular health; SB increases susceptibility to CVD.	Education Persuasion
**5.3** **(a,b,c,d,e)**	***Information about social and environmental consequences***	Provide information (e.g., written, verbal, visual) about social and environmental consequences of performing the behaviour	Inform person about benefit of engaging in non-sedentary activity/ movement with children grandchildren/ pets/ friends (social benefit).	Education Persuasion
**5.6** (a*,b,c,e)	***Information about emotional consequences***	Provide information (e.g., written, verbal, visual) about emotional consequences of performing the behaviour	Explain (e.g., verbally, provide leaflets, by video) that SB is linked to anxiety/ depression and non SB is related to reduced anxiety/ depression/ happiness	Education Persuasion
**6.1** **(a,b,c,e)**	***Demonstration of the behaviour***	Provide an observable sample of the performance of the behaviour, directly in person or indirectly, e.g., via film, pictures, for the person to aspire to or imitate (includes ‘Modelling’)	Demonstrate to the person how to increase energy expenditure in sitting, e.g., weights, bands, pedalling, chair activities.	Training Modelling
**7.1** **(a,b,c,d,e)**	***Prompts/ cues***	Introduce or define environmental or social stimulus with the purpose of prompting or cueing the behaviour. The prompt or cue would normally occur at the time or place of performance.	Put a sticker on the remote control to remind person to delay tv time/ take a movement break when changing channel. Put an auditory sensor mat in front of sofa or sitting room to remind person to delay sitting	Education Environment restructure
**8.1** **(a,b,c,d,e)**	***Behavioural practice/ rehearsal***	Prompt practice or rehearsal of the performance of the behaviour one or more times in a context or at a time when the performance may not be necessary, in order to increase habit and skill	Prompt person to practice seated movements	Training
**9.1** **(a,b,d,e)**	***Credible source***	Present verbal or visual communication from a **credible source** in favour of or against the behaviour	Present a speech given by a PwO or obesity specialist/ HCP to emphasise the importance of non SB for PwO.	Persuasion
**11.2** (a*,b,c,e)	***Reduce negative emotions***	Advise on ways of reducing negative emotions to facilitate performance of the behaviour (includes ‘**Stress Management**’)	Advise on the use of stress management skills, e.g., breathing exercises and mindful movement to reduce feeling incapable of non SB or that it is worthless. Or internal bias.	Enablement
**12.1** **(a,b,c,d,e)**	***Restructuring the physical environment***	Change, or advise to change the physical environment in order to facilitate performance of the wanted behaviour or create barriers to the unwanted behaviour (other than prompts/ cues, rewards and punishments)	Advise to keep screens/ ipads/ phone/ remote control in an inconvenient place. Advise person to use/ change seating to easy to stand from seats.	Enablement
**12.5** **(a,c,d,e)**	***Adding objects to the environment***	Add objects to the environment in order to facilitate performance of the behaviour	Provide pedals/ resistance bands/ pedometer, mobility aids.	Enablement
**12.6** (a*,b,e)	***Body changes***	Alter body structure, functioning or support **directly** to facilitate behaviour change	Prompt strength training, balance training or provide assistive aids (e.g., mobility aid, aids for seated activity)	Enablement
**13.2** (a*,d,e)	***Framing/ reframing***	Suggest the deliberate adoption of a perspective or new perspective on behaviour (e.g., its purpose) in order to change cognitions or emotions about performing the behaviour (includes ‘**Cognitive structuring**’).	Suggest that the person might think of the tasks as maintaining mobility and ability rather than reducing sedentary behaviour (or losing weight/ increasing PA)	Persuasion Enablement
**13.4** (a*,e)	***Valued self-identity***	Advise the person to write or complete rating scales about a cherished value or personal strength as a means of affirming the person’s identity as part of a behaviour change strategy (includes ‘Self-affirmation’)	Advise the person to write about their personal strengths before they receive a message advocating for movement breaks, reduced SB	Enablement
**15.1** (a*,b,c,d,e)	***Verbal persuasion about capability***	Tell the person that they can successfully perform the wanted behaviour, arguing against self-doubts and asserting that they can and will succeed	Tell the person that they can successfully decrease their SB, despite their pain and bodyweight.	Persuasion Enablement
**15.4** (a*,c,d,e)	***Self-talk***	Prompt positive self-talk (aloud or silently) before and during the behaviour	Prompt the person to tell themselves that all movement matters and/ that or a movement break will be energising, will be worth it, is maintaining mobility, enhancing health.	Training Enablement

Source: a = BCW (most frequent); a* = BCW (less frequent); c = TDF mapped to BCW table 3.4; d = scientific literature; e = Research team contextual discussion.

#### Step 8: identify mode of delivery, and measurement of outcomes.

***8(a) mode of delivery and intervention features.*** Modes of delivery are presented for each intervention function and related BCTs (Table 4 and see Table S4 in S1 File).

**Table 4:** Intervention features and content suggested by people with obesity.

Intervention Functions and suggested content, mode of delivery and style of delivery			
Intervention function and chosen BCT’s	Mode of delivery	Suggested content	Style of delivery
Education BCT’s 2.2, 2.3, 5.1, 5.3, 5.6, 7.1 Training BCTs 2.2, 2.3, 4.1, 6.1, 8.1, 15.4 Persuasion BCTs 2.2, 5.1, 5.3, 5.6, 9.1, 13.2, 15.1 Enablement BCTs 1.2, 1.4, 2.3, 3.1, 3.2, 11.2, 12.6, 13.2, 13.4, 15.1, 15.4	adapted exercises at home; virtual/online class; in an email or print (place on wall) Virtual walks active gaming Group PwO, exercise group/class for PwO, adult only, Webinars Awareness Ad campaign, flyers, magazines, social media, TV, Population level awareness, Media campaign target SB/ movement; Slogan (advertising), social media (Instagram); prompts, vibration, feedback (some dislike) Self-monitoring diary	improve ability, belief re ability coping skills for pain and lack of ability, Pacing/boom bust alternatives, achievable steps, slower, movements/ exercise, 100 steps X 10 ad breaks, 2–10 x sit/ stand, buttock clenches, adapted Zumba/ aqua fit, Yoga, Soft tennis, step for push up, Balance exercises at home, Body realignment, gait retraining, chair exercise, yoga, pedals, exercise for purpose, orienteering, family orienteering, simple exercises, program, graded, standing, chair, resistance, strength, weights, flexibility, at home, Just getting up, Just mobility. education of PwO, HCP, GP, Providers care/intervention, public, school re complexity, factors influencing SB, occupational SB, re obesity (as disease, complexity, treatments, care, setpoint) re access to supports, to adapt exercises, re health consequences (SB, Obesity) re health benefits (Non SB, LPA, comfort breaks daily mobility, empathetic) re home movement, staying mobile, Self-awareness, re need for change acceptance, internalised bias PwO,	not didactic, progress not perfection,

***Mode of Delivery of education BCT’s 2.2, 2.3, 5.1, 5.3, 5.6, 7.1.*** Participants recognised the need for education about physical, health and psychological benefits or consequences of non-SB and SB. Most identified a need for education about the complexity and interconnection of obesity, movement, nutrition, SB, sleep and mental health, while information about access to supports and services was also considered important. Participants suggested online delivery of information (BCT’s 5.1, 5.3,5.6).

*“We got on webinars and knowing that you’re not on your own. It’s not just you like you know that, and it’s not my fault. That’s even more important” PID007*

*“Maybe just to put it lightly to people that there is a good benefit. I don’t know. I wouldn’t have a scientific understanding of what are the benefits of moving around or whatever. But I think if the facts were laid bare to somebody, just in a nice way, that it might just twig something in their brain to purposely do something” PID001*

Monitoring/ self-monitoring and prompts and cues (BCTs 2.2, 2.3 7.1) was mentioned by many participants, either via a wrist worn device, mobile app or diary. Vibrotactile or audible prompts and cues, were mentioned as possible features of an intervention but some participants believed this could elicit negative feelings and be demotivating. Optional use of this feature may be an important consideration for the intervention. Alternative prompts could be more acceptable to some people with obesity, e.g., ad-breaks on TV or radio.

*“I used to have a thing on my iWatch that used to vibrate if I was sitting for too long, if I wasn’t moving for too long. ….It was great because it would just remind you. But you see, for me, at times, say you’re standing in the one spot ….. It would then vibrate and you’re like, “Oh my God, I can’t move...” Just moving your legs in that would help” PID005*

***Mode of Delivery of training BCTs 2.2, 2.3, 4.1, 6.1, 8.1, 15.4.*** To deliver training BCTs related to behavioural feedback, instruction, demonstration, practice, persuasion about capability (2.2,4.1,6.1,8.1,15.4) most participants requested some type of exercise or movement rather than simply standing, and although some mentioned exercises that could be emailed or put on a wall, most wanted virtual classes, delivered weekly by skilled and encouraging coaches or health professionals who had empathy for and experience with people living with obesity. Participants also recognised the need for psychological skills and support to retrain thought processes to value non-SB versus physical activity.

*“If you gave maybe some exercises, if there were some in an email or something they could print out, maybe put it on a wall” PID021*

*“At least in your own house, you can sit down if you’re breathing, or if you start to cough, sometimes you can get a coughing spasm with breathlessness. So I think maybe if people were aware that there are classes online on YouTube, or if the HSE (health service executive) were to start classes, I think it’d be great” PID008*

*“What’s the point? You’re not even breaking a sweat….. So it’s like, I do need to retrain my thoughts around that. The benefits of just moving, even if I don’t be breathless at the end of it. Yeah” PID013*

The ability to remain anonymous and have the camera off during virtual movement or education classes was an important feature for people with obesity.

*“Yeah, I think that some people want to remain anonymous.... There’s some people that don’t turn on the camera because they’re camera shy. Or they don’t want people to see them because of their size, or because they’re struggling with their identity. Or they might not even want to look at themselves on the screen. I think, yeah, unfortunately” PID008*

***Mode* of *Delivery* of *enablement (BCTs 1.2, 1.4, 2.3, 3.1, 3.2, 11.2, 12.6, 13.2, 13.4, 15.1, 15.4).*** Engagement with other people with obesity, in online classes, or social media whereby people could feel ‘normal’ was considered important and suitable to deliver enablement BCTs related to social support (3.1, 3.2) body changes (12.6) identity and self-belief (13.2, 13.4, 15.1, 15.4). So too was timely skilled human intervention via phone or online, to deal with negative emotions (BCT 11.2) particularly attached to weight, weight stigma, poor nutritional choices, or lack of physical activity or motivation.

*“I suppose, when I think of like, I don’t know what you could put in place, but for example, for me, that phone call broke it for me. It’s some form of support to realise that just because you fall down on one piece doesn’t mean you have to lose it all” PID009*

*“A facility, for example, that the weight management clinic has is the physio there, does online movement classes for graduates of the program. That’s accessible movement, and it’s safe and it’s guided, and you get the companionship of other people, etc. etc. and it’s kind of normal. Some people can stand up, some people can’t stand up” PID018*

No mode of delivery for goals and planning (BCT 1.2, 1.4) was identified, although participants had mixed view regarding the inclusion of these BCTs since some many had negative experiences of goalsetting regarding weight loss or physical activity.

*“I’d like to write down why I didn’t make that goal or what were the factors that contributed to it, and then maybe look at that and focus on those to try to change that. PID012*

*The fact that I’m not setting hard goals for myself to fail, then I know I’m doing my best for now” PID003*

***Mode* of *Delivery* of persuasion (BCTs 2.2, 5.1, 5.3, 5.6, 9.1, 13.2, 15.1).** Participants considered that a credible source (9.1) is essential to delivery of any intervention delivered in via print, or online. Credible sources included other people with obesity, trained and skilled coaches or health professionals. All other BCT’s for persuasion overlap with intervention function discussed above.

*“For one, I think listening to like say, for example, … someone who was maybe used to dealing with obese people. Because I would say, look, a lot of trainers at gyms and things who go and do courses, but I don’t think they have a full understanding of again, that lack of ability to move” PID009*

*“He started this online thing and I went back to doing his stuff. He’s not just a PT where he’s just trying to make you thin. He’s very much about your movement and there’s always kind of a 20 minutes stretchy part, as well as the strength stuff, as well as a bit of cardio stuff. He can be very good like that” PID015*

#### Further intervention features.

Participants recognised the difficulty with branding a class for people with obesity and were concerned that it could perpetuate weight stigma and thereby be unacceptable. Age appropriateness was also considered important although classes suitable for seniors were mentioned as suitable for people with obesity but not desirable for younger people with obesity. Fun, enjoyment, and music were also mentioned as important features.

“*That was really good that was on Zoom. I can’t remember what it was though, but it was free and it was very good. But again, it was geared towards seniors. It’s great that there’s classes for seniors, but I don’t think there should be an age on it. Because, I was telling a friend who’s also heavy and she was like, for sure, that’s for older people. I told her, no, I actually emailed the lady who’s doing the class and she said you could join in” PID008*

#### Style of delivery.

Regarding style of delivery, language used and empathy were considered crucial. The term ‘behaviour change’ elicited negative feelings for some as did being told what to do rather than encouraged and having choice.

*“Now I like, I would have huge issues… when they say, you know patients need behaviour change and it goes behaviour change, behaviour change through the whole document. I’m like, “Can you not put in your routines or your habits or change the wording? Because I’m starting to feel like you’re giving out to me about my bad behaviour. I’m not a child. I know, you know, what I need to do” PID003*

*“So it’s about meeting somebody wherever they are and just really giving them that little bit of encouragement to stand beside them while they try and do any little bit that they can” PID015*

#### 8(b) Measurement of outcomes.

Measurement and reporting of outcomes will be guided by the core outcome set for SB interventions developed in work stream 2 and reported elsewhere [41]. This core outcome set specifies what, when and how to accurately measure and quantify SB. It consists of 53 data items and data domains related to demographics, device details, wear-time criteria, wear-time measures, posture-related measures, sedentary breaks, sedentary bouts and physical activity. A key recommendation regarding the functionality of devices, is the use of an accelerometer with an inclinometry function to distinguish the postural components of SB, sitting or reclining vs standing.

### 3.4 Development of guiding principles

Finally, to complete this PBA phase of the intervention development, guiding principles were created by collating the above steps, by stating the objectives of the intervention in terms of behaviour and outcomes, summarising relevant aspects of users and their context and identifying key behavioural issues, needs, or challenges the intervention must address. Thus, the key objective of the intervention is to support people living with obesity to reduce their leisure time sedentary behaviour at home.

#### Contextual relevance and key behavioural challenges summary.

People living with obesity may have poor physical ability, co-morbidity, or mental health which contributes to SB and damaged identities due to weight bias/stigma. Beliefs about their capabilities or competing beliefs about need for PA, diet, weight loss influence SB, while limited knowledge and poor value on non SB will also need to be addressed. Human, social and practical support for movement, delivered via telehealth, including physical and psychological skills, counter-narratives, and peer support to normalise experiences, motivate and enhance non-SB may improve functional ability, skills, and beliefs about capabilities. Some resistance to the word ‘behaviour change’ while referring to sedentary behaviour with obesity may perpetuate internal and external stigma, therefore adopting ‘Make all movement matter’ approach may be more effective.

People with obesity may lack confidence, skills or ability to engage in consistently non-SB, have negatively internalised the ‘eat less move more’ message while feeling shame and embarrassment that despite eating less and moving more they continue to live with obesity. Thus, movement not attached to weight loss may have little value, while body size, pain and physical effort affect beliefs about capabilities. Guiding principles and intervention content and strategy are detailed in Table 5.

**Table 5:** Intervention content, strategy and guiding principles.

Guiding principles	Key Objectives To help people living with obesity to develop and/ or maintain non-sedentary behaviour at home in the long term. To assure people living with obesity that non-sedentary movement is safe for and of value to them, and to promote self-efficacy for non-sedentary behaviour.		Key features Telehealth/ Online intervention to build autonomous motivation, physical and psychological skills (e.g., education, self-monitoring, problem solving) and confidence (beliefs about capabilities) to become their own movement coach. Focus on creating sustainable non-sedentary habits and beliefs, value movement for function and quality of life, independent of weight/ body size/ rather than relying on supervised activities. Encouragement for and normalisation of non-sedentary behaviours provided in terms of obesity and co-morbidity including specific advice on benefits of movement, engaging with other PwO in online class, independent of physical activity.			
**Intervention functions**	**TDF Domain**	**COM-B components**	**BCTs to deliver intervention functions**		**Policy categories**	**Intervention strategy**
Education	Knowledge Beliefs	Capability Motivation (automatic/ reflective)	2.2	*Feedback on behaviour*	Communications, Guidelines, Service provision	Initial period of device measured SB with self-monitoring (to include inclinometer function), parallel self-monitoring diary and provide feedback, prompts and cues. Self-monitoring diary to continue periodically, e.g., 2 days/ week. Information about consequences (health social environmental and emotional, delivered by HCP movement coach in person/ by telehealth + /- written format
			2.3	*Self-monitoring of behaviour*		
			5.1	*Information about health consequences*		
			5.3	*Information about social and environmental consequences*		
			5.6	*Information about emotional consequences*		
			7.1	*Prompts/ cues*		
Training	Skills (Physical/ Psychological)	Opportunity (Physical/ Psychological)	2.2	*Feedback on behaviour*	Communications, Guidelines, Service provision	Initial period of device measured SB with self-monitoring (to include inclinometer function), parallel self-monitoring diary and provide feedback, prompts and cues. Self-monitoring diary to continue periodically, e.g., 2 days/ week. Instruction, demonstration and practice of behaviour and appropriate self-talk/ taglines delivered by HCP movement coach by telehealth via movement and skills class with individual or group PwO + /- written format
			2.3	*Self-monitoring of behaviour*		
			4.1	*Instruction on how to perform a behaviour*		
			6.1	*Demonstration of the behaviour*		
			8.1	*Behavioural practice/ rehearsal*		
			15.4	*Self-talk*		
Persuasion	Emotion,	Motivation (automatic)	2.2	*Feedback on behaviour*	Communications, Guidelines, Service provision	Initial period of device measured SB with self-monitoring (to include inclinometer function), parallel self-monitoring diary and provide feedback, prompts and cues. Self-monitoring diary to continue periodically, e.g., 2 days/ week. Information about consequences (health social environmental and emotional, delivered by HCP movement coach by telehealth via movement and skills class with individual or group PwO. Counter narratives delivered by HCP movement coach as above by telehealth, may include video-stories
			5.1	*Information about health consequences*		
			5.3	*Information about social and environmental consequences*		
			5.6	*Information about emotional consequences*		
			9.1	*Credible source*		
			13.2	*Framing/ reframing*		
			15.1	*Verbal persuasion about capability*		
Enablement	Knowledge, Memory, Awareness And Decision Processing, Behavioural regulation	Psychological Capability	1.2	*Problem solving*	Communications, Guidelines, Service provision	Initial period of device measured SB with self-monitoring (to include inclinometer function), parallel self-monitoring diary and provide feedback, prompts and cues. Self-monitoring diary to continue periodically, e.g., 2 days/ week. Social support delivered via telehealth movement and skills class with individual or group PwO. To include movement practice, planning, problem solving. Counter narratives delivered by HCP movement coach as above by telehealth
			1.4	*Action planning*		
			2.3	*Self-monitoring of behaviour*		
			3.1	*Social support (unspecified)*		
			3.2	*Social support (practical)*		
			11.2	*Reduce negative emotions*		
			12.6	*Body changes*		
			13.2	*Framing/ reframing*		
			13.4	*Valued self-identity*		
			15.1	*Verbal persuasion about capability*		
			15.4	*Self-talk*		
Environmental restructuring	Beliefs Intentions	Capability (physical) Opportunity (physical/ social)	7.1	*Prompts/ cues*	N/A domestic environment	Use of devices to deliver the above functions
			12.1	*Restructuring the physical environment*		
			12.5	*Adding objects to the environment*		
Modelling	Knowledge	Physical capability	6.1	*Demonstration of the behaviour*	Communications, Service provision	Included above

## Discussion

This paper describes the systematic development process of a theory, evidence and person-based intervention to reduce SB in the domestic domain for and with people living with obesity. Identifying appropriate theoretical content, (behaviour change techniques and theoretical constructs) ensures that the effective components of behaviour change and effective mechanisms of action can be included in intervention design, thus improving potentially efficacy [35]. Inclusion of the target population to inform modes of delivery and appropriate features provides a context sensitive intervention likely to be acceptable and feasible [37]. To design the intervention, prior formative work included gathering evidence by conducting two systematic reviews, and primary qualitative research with the target population and stakeholders. Rigour was enhanced throughout, by comprehensive application of the BCW including mapping to the COM-B model, TDF and the use of the behaviour change taxonomy (BCTv1) and theory and techniques tool, to underpin formative work and subsequently select theoretical content. Additionally, modes of delivery and intervention features were identified and mapped to the intervention functions of education, training, persuasion and enablement, resulting in design of a theory, evidence and person-based intervention and development of a set of guiding principles, aligned to the PBA.

This is the first study to date to utilise this comprehensive approach to SB intervention development targeting the domestic domain or with a population of people living with moderate to severe obesity, although similar approaches have been used to develop SB interventions in the workplace [26,30] and with a clinical population [51]. This work provides further detail regarding potentially effective interventions components and SB examples for all BCTs, based on the best evidence to date. The use of the BCW theoretical framework is highly advantageous, providing a standardised terminology for constructs and components of interventions (e.g. BCTs, intervention functions), thereby improving both replicability and synthesis of evidence [47], and maximising the potential benefit of future SB interventions. Further work is required to test the intervention described here.

While motivation to change SB may be low for some people with obesity, our qualitative research identified that many are highly motivated to change SB, to maintain and improve functional ability particularly following a physical or mental health crisis, but that education, persuasion, training and enablement may be necessary. This contrasts with participants in workplace domains, where motivation to change SB may be low due to workplace culture and habit [30,52]. Nonetheless, similar intervention functions may be chosen to target SB change, while BCTs and intervention features and modes of delivery may be quite different. Similar to prior research, simply asking people to stand up was not perceived as acceptable [52] nor is it likely to be sustained, albeit for different reasons in people with obesity, such as experiences of chronic pain. SB breaks which include purposeful movement or increased energy expenditure in sitting were considered more desirable and an important measure in sustaining or recovering health and wellbeing.

The secondary analysis of qualitative data informed the choice of intervention content and features, and furthermore provides guidance for future researchers and clinicians regarding their approach to the population of people with obesity. The approach suggested by participants in the qualitative analysis is aligned with prior work with this population, which uses the 5As (ask, assess, advise, agree/arrange, and assist) framework of obesity management [53–55]. Assessing and respecting the readiness or resistance to behaviour change was highlighted by the participants. Sedentary behaviour is a sensitive issue for people with obesity who may feel judged and stigmatised or may not be ready to change it. Regarding the intervention function of education, participants wanted to know about SB but needed to be asked if they would like to talk about sitting or moving and encouraged empathetically, rather than assumed to be sedentary or told what to do. Similar to workplace SB interventions [Munir Edwardson], knowledge or awareness alone was not enough to shift intrinsic motivation to action. Reframing the target behaviour in terms of mobility recovery or maintenance, enhanced motivation for people with compromised mobility, similar to studies of people with osteoarthritis [56]. Moreover, the participants also highlighted the importance of the function of enablement, by following up with assistance or arranging access to appropriate supports or services which they deemed necessary to facilitate SB change, a potential gap in service provision for people with obesity.

Although not developed here, brief intervention counselling targeting SB change rather than physical activity may be appropriate for some people with obesity who lack, or perceive that they lack the capability or opportunity to engage in PA. Some people with obesity may perceive little benefit to standing or light PA, but described a negative cycle comprised of not achieving PA or weight loss, negative feelings, increased SB, poorer nutritional choices, thereby repeating the cycle. Empathetic engagement about the benefit of non-SB, may thus enhance the therapeutic encounter and be sufficient to motivate people with obesity to maintain or enhance their physical and mental wellbeing. However, clinicians and researchers must not make the mistake of assuming that any individual person with obesity engages in prolonged SB, as to do so could jeopardise the encounter or therapeutic relationship by perpetuating the stigmatised stereotype. Further research to identify the potentially effective components for this type of encounter is necessary [55].

Notably, although the behavioural diagnosis is intended to highlight the constructs which most need to change, similar to other research which used the BCW framework, barriers were identified across all domains of the COM-B and TDF [51]. Thus, all intervention functions and policy categories in the BCW were potentially relevant, and pragmatic decisions were made using the APEASE criteria [33]. Many opportunities exist to influence SB in the population of people with obesity which are beyond the scope of this work. Arguably, urgent attention to the barriers and behaviours beyond the individual level may be crucial to the health outcomes of people with obesity, for example, education of healthcare practitioners regarding obesity bias and stigma to ensure people with obesity feel safe in disclosing their SB or that there are supports accessible and available.

## Strengths and limitations

The strength of this study lies in the robust and transparent process provided by use of the BCW, TDF, COM-B and theory and techniques tool in tandem with the evidence gathered in two robust systematic reviews [13,24], a qualitative study [25] and a Delphi study [44]. Aligned to the MRC framework, this deeply contextualises the evidence and theory to the target population and setting. This phase of intervention development is lengthy, and although from a pragmatic perspective, the intervention was not further developed or tested, by making the theoretical content available for future intervention design, future researchers can move swiftly to locally contextualised co-development, feasibility testing and randomised trials [57]. While data was limited on style of delivery, further development of the style and features of development in local context will enhance acceptability of the intervention aligned with the recently developed ontology [58]. (See Table S9 in S1 File). Indeed, as advised in the BCW [33] all 93 BCTs were considered for the intervention and a SB example was developed for each, a valuable tool for SB researchers. (See Table S8 in S1 File)

However, some limitations exist. Firstly, the intervention development targeted individual level behaviour change but not the behaviour of providers of social, environmental or political supports, including healthcare providers. Undoubtedly, as identified in prior research, barriers to behaviour change for people with obesity exist at each of these levels [13,25]. Indeed, without the system level changes required to comprehensively support and treat obesity and its co-morbidities, people with obesity may lack the capability or opportunity to meaningfully change their SB beyond the domestic domain. Secondly, although the intervention development pertains to the domestic domain for people with obesity, the BCTs identified have not necessarily been tested in this domain, or with this population. They do however, represent the best current evidence for sedentary behaviour change and aligned to contextualised person-based development, are most likely to be effective, and to facilitate future evidence synthesis. Furthermore, the original qualitative study which informed the selection of contextualised components of the intervention [25] included both male and female participants and a wide age range (31–64 years). However, it also included many participants who had prior bariatric surgery, and therefore may not be representative of all people with obesity. However, this may be considered both a strength and limitation, since their experiences and perspectives generally included the entire spectrum of the disease of obesity, from development, through multiple cycles of relapse and remission, and the corresponding association with SB. The complexity of reporting a multi-study, multiple framework program necessarily limits how fully the rigour and overall robustness of the work an be detailed. Finally, the development process was protracted due to limitations in evidence requiring further research which ultimately strengthen the work. However, the BCTv1 taxonomy is now superseded by the behaviour change ontology [59,60] and indeed rapid progress in artificial intelligence will undoubtedly advance the accumulation of evidence. It is imperative therefore, to explicitly integrate theory into the design, implementation and reporting of interventions. This will ensure that machine learning models are built on meaningful, high quality inputs and return high quality outputs.

## Conclusions

This paper describes the systematic development process of a theory, evidence, and person-based intervention to reduce leisure time sedentary behaviour with and for people with obesity in the domestic domain. The transparent and robust process, utilising the BCW, TDF and COM-B to select the intervention components (features, BCTs and potential mode of delivery), resulted in identification of 22 BCTs with potential to reduce SB for this population. Further potential modes and styles of delivery, and supportive policies are identified which are grounded in qualitative discussion with people with obesity. The work presented here provides a guide to intervention development and the evidence, theory and person-based foundation for co-design or co-production of interventions likely to change SB, particularly for but not limited to people with obesity, and thereby reduce the lengthy first phase of the MRC complex intervention development for future researchers who apply these resources in their specific context.

## Supporting information

S1 File S1 Supporting information. (DOCX)
