# Supplementary material for: Developing theory and evidence based intervention content using the Behaviour Change Wheel, Theoretical Domains Framework, and the Person-Based Approach: A worked example for an intervention targeting sedentary behaviour with people living with obesity
Source: PLoS One. 2025 Dec 5;20(12):e0338196. doi: 10.1371/journal.pone.0338196 (PMC12680251; doi:10.1371/journal.pone.0338196)
Supplement: S1 File — (DOCX) [file pone.0338196.s001.docx]

Supporting Information File

*Developing theory and evidence based intervention content using the Behaviour Change Wheel, Theoretical Domains Framework, and a person-based approach: a worked example for an intervention targeting sedentary behaviour with people living with obesity (PwO)*

**List of Tables**

Table S1. *Define the problem in behavioural terms*

Table S2. *Select the target behaviour*

Table S3*. Identify what needs to change*

Table S4*:* *Identify what needs to change using the Theoretical Domains Framework (TDF)*

Table S5: *Intervention functions APEASE*

Table S6*: Identify policy categories*

Table S7: *BCT’s with SB examples for all 93 BCTs*

Table S8: *Longlist BCT’s with potential to change SB*

Table S9: *Intervention features and mode of delivery suggestions mapped to TDF COM-B*

**Table S1.** Define the problem in behavioural terms

| **What behaviour?** | **Prolonged Sedentary Behaviour** | **Potential for intervention** |
| --- | --- | --- |
| **Where does the behaviour occur?** | Domestic domain (home) | Good potential at individual level |
|  | Occupational domain | Not practical (may not reach target PwO) |
|  | Transportation domain | Not practical (physical /environmental barriers) |
|  | Community domain | Some potential, (environmental, psychological barriers) |
| **Who is involved in performing the behaviour?** | People with obesity |  |
| **When does the behaviour occur?** | Leisure time | Promising (can reach all PwO) |
|  | Work time | Not practical (not all PwO in work) |
|  | During other responsibilities | Not practical (too varied) |

**Table S2***.* Select the target behaviour

| **Intervention Aim: Reduce / minimise SB in leisure time at home PwO** | | | | | Decision |
| --- | --- | --- | --- | --- | --- |
| **Candidate target behaviours that could reduce SB at home / Leisure time** |  |  |  |  |  |
| Physical activity |  |  |  |  | out ruled by problem statement |
| **Sedentary Behaviour** (components) |  |  |  |  | target |
| **What** | **Who** | **When** | **Where** | **With whom** |  |
| **Posture** | PwO | every 30-60 min / daily | at home /domestic | alone / not dependent on others | include as practical and affordable |
| • Change posture to standing |  |  |  |  |  |
| • Add movement breaks (standing and moving) |  |  |  |  |  |
| **Energy** | PwO | every 30-60 min / daily | at home /domestic | alone / not dependent on others | include as add movement break (seated /reclined) |
| • Increase energy expenditure in sitting / reclining / lying |  |  |  |  |  |
| **Awake** |  |  |  |  |  |
| • Encourage Sleep | PwO | meet sleep guidelines / 8/hours | at home /domestic | alone / not dependent on others | Possible inclusion as part of education; targeted alone not likely to change SB enough |

**Table S3*.*** Identify what needs to change

| **COM-B  Components** | **What needs to happen for the target behaviour to occur?** | **Is there a need for change?** |
| --- | --- | --- |
| Capability: physical | Be able to stand /move relatively easily  (ability, strength, stamina) | Change needed |
| Capability: psychological | Know that SB should be broken every 10 -30 minutes / minimised | Change needed |
|  | Understand / believe that breaking SB is possible and beneficial with respect to pain / ability |  |
|  | Know how to establish reminders, routines and habits to stand / take movement break |  |
| Opportunity: physical | Have seating that is easy to rise from or aids to stand / move available | Change needed |
|  | Have devices for seated activity |  |
|  | Have home screens usable from standing positions |  |
| Opportunity: social | Have access to support to develop physical and psychological skills for standing and moving easily (one to one; group) | Change needed |
|  | Be socially comfortable moving in the home environment when others present. |  |
| Motivation: reflective | Hold the belief that if they break SB / stand / move, it will maintain / improve mobility, physical or mental health. | Change needed |
|  | Intend to and prioritise standing and movement breaks |  |
| Motivation: automatic | Establish routines and habits to stand or take movement breaks | Change needed |
| **Behavioural diagnosis:** | | |
| Physical and psychological capability, physical and social opportunity,  and reflective and automatic motivation need to change in order for the target behaviour 'standing up from sitting / reclining’ or ‘take movement breaks' to occur. | | |

**Table S4.** Identify what needs to change using the Theoretical Domains Framework (TDF)

| **COM-B and TDF relevance to behavioural diagnosis.** | | | |
| --- | --- | --- | --- |
| **COM-B component** | **TDF Domain** | **Theoretical constructs** | **Relevance of domain**  **(what needs to change)** |
| Physical capability | **Physical skills** *An ability or proficiency acquired through practice* | Skills  Skills development Competence Ability Interpersonal skills Practice  Skill assessment | Have the physical skills, strength and stamina to stand or move from reclining / sitting (consider pain, mechanical and medical barriers) or perform seated movements |
| Psychological capability | **Knowledge**  *An awareness of the existence of something* | Knowledge (including knowledge of condition / scientific rationale). Procedural knowledge. Knowledge of task environment | Know what non-SB is and how to break it. Know how to create and apply 'if- then' rules to prompt non SB (standing/ movement breaks) Know association of SB, obesity, mental, physical health, sleep, nutrition, PA. |
|  | **Cognitive and interpersonal skills** *An ability or proficiency acquired through practice* | Skills  Skills development Competence Ability Interpersonal skills Practice  Skill assessment | Be able to prioritise and advocate for personal wellbeing / non SB in the familial / home context (e.g. reframe comfort breaks / tasks and chores / caregiving) |
|  | **Memory, attention and decision processes** *The ability to retain information, focus selectively on aspects of the environment and choose between two or more alternatives* | Memory Attention Attention control Decision making Cognitive overload / tiredness | Notice and remember to stand or take a movement break Believe that non SB is valuable to health independent of PA, body weight, nutritional status  Be able to choose / prioritise non SB over competing behaviours, (particularly when demotivated, tired) |
|  | **Behavioural regulation** *Anything aimed at managing or changing objectively observed or measured actions* | Self-monitoring Breaking habit Action planning | Develop skills of goal setting, self- monitoring and action planning and applying 'if-then' rules |
| Physical opportunity | **Environmental context and resources** *Any circumstance of a person's situation or environment that discourages or encourages the development of skills and abilities, independence, social competence, and adaptive behaviour* | Environmental stressors Resources / material resources  Organisational culture /climate Salient events / critical incidents Person x environment interaction Barriers and facilitators | Use seating which is easy to stand from,  or equipment to increase energy expenditure when using screens (e.g. standing during screentime or seated PA equipment) Provide materials / resources which demonstrate / develop skills for, non-SB |
| Social opportunity | **Social influences** *Those interpersonal processes that can cause individuals to change their thoughts, feelings, or behaviours* | Social pressure Social norms Group conformity Social comparisons Group norms Social support Power Intergroup conflict Alienation Group identity Modelling | The opportunity to engage / practice non SB with peers PwO and HCPs skilled and informed in the complexity of obesity and the barriers to non-SB. |
| Reflective motivation | **Social / professional role and identity** *A coherent set of behaviours and displayed personal qualities of an individual in a social or work setting* | Professional identity  Professional role  Social identity  Identity  Professional boundaries Professional confidence  Group identity Leadership Organisational commitment | Develop identity as non-sedentary adult, (independent of past current identity as 'physically active / inactive', 'sporty' or 'lazy')  Be able to identify SB in competing social / familial roles, Be able to boundary professional / voluntary activities at home |
|  | **Beliefs about capabilities** *Acceptance of the truth, reality, or validity about an ability, talent, or facility that a person can put to constructive use* | Self-confidence  Perceived competence  Self-efficacy Perceived behavioural control  Beliefs  Self-esteem  Empowerment  Professional confidence | Believe that non-SB is possible despite some limitations (physical / medical / pain / body weight). Believe that consistent non-SB will require improved cognitive and self-regulation skills. |
|  | **Optimism** *The confidence that things will happen for the best or that desired goals will be attained* | Optimism  Pessimism Unrealistic optimism Identity | Develop confidence that non-SB is achievable and worthwhile independent of weight / PA / obesity  Reduce unrealistic optimism of weight loss |
|  | **Beliefs about consequences** *Acceptance of the truth, reality, or validity about outcomes of a behaviour in a given situation* | Beliefs Outcome expectancies Characteristics of outcome expectancies Anticipated regret Consequents | Believe that consistent non-SB will benefit physical and psychological health independent of body weight/ obesity Believe that SB contributes to deterioration in physical and mental health and obesity. Believe that non-SB has health benefits / consequences independent of PA / obesity / nutritional intake |
|  | **Intentions** *A conscious decision to perform a behaviour or a resolve to act in a certain way* | Stability of intentions Stages of change model Trans-theoretical model and stages of change | Develop intention / decision to interrupt SB |
|  | **Goals** *Mental representations of outcomes or end states that an individual wants to achieve* | Goals (distal / proximal) Goal priority Goal / target setting Goals (autonomous / controlled) Action planning Implementation intention | Set realistic achievable (smart) goals, independent of past or future / failures |
| Automatic motivation | Reinforcement *Increasing the probability of a response by arranging a dependent relationship, or contingency, between the response and a given stimulus* | Rewards (proximal / distal, valued / not valued, probable / improbable) Incentives Punishment Consequents Reinforcement Contingencies Sanctions | Reduce negative reinforcement associated with non SB especially pain (pacing vs boom bust) Reinforce non sedentary routines and habits |
|  | **Emotion** *A complex reaction pattern, involving experiential, behavioural, and physiological elements, by which the individual attempts to deal with a personally significant matter or eve*nt | Fear Anxiety Affect Stress Depression Positive / negative affect Burn-out | Reduce (negative emotion) associated with non SB at home fear of movement / falling / judgement / pain / non achievement. Promote acceptance of self /current ability body / obesity  Identify enjoyable non-SBs  Encourage awareness of affect on SB and non SB Develop awareness and coping skills for negative emotions particularly internal bias |

**Table S5.** Intervention functions APEASE

| **Candidate intervention functions** | **Is the candidate intervention function relevant, affordable, acceptable, practicable, likely to have impact in this context?** |
| --- | --- |
| Education | Yes |
| Persuasion | Yes |
| Incentivisation | Not practicable or affordable |
| Coercion | Unlikely to be acceptable |
| Training | Yes |
| Restriction | Not practicable or relevant |
| Environmental restructuring | Yes |
| Modelling | Yes |
| Enablement | Yes |
| **Intervention functions chosen**  Education; Persuasion; Training; Enablement;  + /- Environmental restructuring; Modelling; | |

**Table S6.** Identify policy categories

| **Intervention function** *definition* | **Policy categories that could deliver intervention functions** | **Does the policy category meet the APEASE criteria (affordability, practicability, effectiveness / cost-effectiveness, acceptability, side- effects/safety, equity) in the context of non SB for PwO in the home?** |
| --- | --- | --- |
| **Education** *Increasing knowledge or understanding* | Communication/marketing | Targeted, well-chosen multimedia education could reach population level |
|  | Guidelines | International SB guidelines available; not specific to PwO |
|  | Regulation | n/a |
|  | Legislation | n/a |
|  | Service provision | Potential role |
| **Persuasion** *Using communication to induce positive or negative feelings or stimulate action* | Communication/marketing | Targeted, well-chosen multimedia education could reach population level |
|  | Guidelines | International SB guidelines available; not specific to PwO |
|  | Regulation | n/a |
|  | Legislation | n/a |
|  | Service provision | Potential role |
| **Incentivisation** *Creating an expectation of reward* | Communication/marketing | Not practicable or affordable |
|  | Guidelines | Not practicable or affordable |
|  | Fiscal measures | Not practicable or affordable |
|  | Regulation | Not practicable or affordable |
|  | Legislation | Not practicable or affordable |
|  | Service provision | Potential role |
| **Coercion** *Creating an expectation of punishment or cost.* | Communication/marketing | Unlikely to be acceptable |
|  | Guidelines | Unlikely to be acceptable |
|  | Fiscal measures | Unlikely to be acceptable |
|  | Regulation | Unlikely to be acceptable |
|  | Legislation | Unlikely to be acceptable |
|  | Service provision | Unlikely to be acceptable |
| **Training** *Imparting skills* | Guidelines | International SB guidelines available; not specific to PwO |
|  | Fiscal measures | potential role in service provision / implementation |
|  | Regulation | n/a |
|  | Legislation | n/a |
|  | Service provision | potential role |
| **Restriction** *Using rules to reduce the opportunity to engage in the target behaviour (or to increase the target behaviour by reducing the opportunity to engage in competing behaviours)* | Guidelines | n/a |
|  | Regulation | n/a |
|  | Legislation | n/a |
| **Environmental restructuring** *Changing the physical or social context* | Guidelines | n/a |
|  | Fiscal measures | n/a |
|  | Regulation | n/a |
|  | Legislation | n/a |
|  | Environmental/social planning | domestic environment n/a |
| **Modelling** *Providing an example for people to aspire to or imitate* | Communication/marketing | Targeted, well-chosen multimedia education could reach population level |
|  | Service provision | potential role |
| **Enablement** *Increasing means/reducing barriers to increase capability (beyond education and training or beyond environmental restructuring)* | Guidelines | International SB guidelines available; not specific to PwO |
|  | Fiscal measures | potential role in service provision / implementation |
|  | Regulation | n/a |
|  | Legislation | n/a |
|  | Environmental/social planning | n/a |
|  | Service provision | potential role |

**Table S7.** BCT’s with SB examples for all 93 BCTs

| BCT No. | BCT Label | Definition | Example For SB reduction | Intervention functions targeted | Frequency  Most M Less L None N BCW (M=22) | TDF link to BCW Table 3.4  (n=43) | T & T tool MoAs (n=38) | Evidence for BCT (n=24) |
| --- | --- | --- | --- | --- | --- | --- | --- | --- |
| 1.1 | ***Goal setting (behaviour)*** | Set or agree a goal defined in terms of the behaviour to be achieved | Agree a daily maximum sitting time or number of movement breaks per hour | Enablement | M | Go,  Q1 | Go,  In, T&T | *Gardner 2015 SLR promise ratio 1.6 |
| 1.2 | ***Problem solving*** | Analyse , or prompt the person to analyse, factors influencing the behaviour and generate or select strategies that include overcoming barriers and/or increasing facilitators (includes ***‘Relapse Prevention’ and ‘Coping Planning’***) | Identify specific triggers (e.g. feeling low/ anxious/tired /pain) that initiate and sustain prolonged sedentary / sitting time and develop strategies for avoiding environmental triggers or for managing e.g. negative emotions, such as anxiety, or physical pain that motivate SB | Enablement | M |  | Br, BaCa, T&T | *Gardner 2015 SLR promise ratio 4.0 |
| 1.3 | ***Goal setting (outcome)*** | Set or agree a goal defined in terms of a positive **outcome** of wanted behaviour | Set a time score e.g. to be able get up from sitting in < 30 seconds, or TUG time or, VAS pain score, or sit stand chair test. | Enablement | M | Go,  Q1 | Go, T&T | *Gardner 2015 SLR promise ratio 2.0 Most potent SLR1 Curran et al 2021 |
| 1.4 | ***Action planning*** | Prompt detailed planning of performance of the behaviour (must include at least one of context, frequency, duration and intensity). Context may be environmental (physical or social) or internal (physical, emotional or cognitive) (includes ***‘Implementation Intentions’***) | Prompt planning taking a movement breaks from evening TV, identifying ad breaks as cues to move, allowing one break to be ignored, move every second break when feeling tired / stressed. | Enablement | M | Go,  Q1 |  | *Gardner 2015 SLR promise ratio 1.7 |
| 1.5 | ***Review behaviour goal(s)*** | Review behaviour goal(s) jointly with the person and consider modifying goal(s) or behaviour change strategy in light of achievement. This may lead to re-setting the same goal, a small change in that goal or setting a new goal instead of (or in addition to) the first, or no change | Examine how well a person's performance corresponds to agreed goals e.g. whether they took SB breaks hourly, and consider modifying future behavioural goals accordingly e.g. by increasing or decreasing SB break targets | Enablement | M | Go,  Q1 | Go, T&T | *Gardner 2015 SLR promise ratio 2.0 |
| 1.6 | ***Discrepancy between current behaviour and goal*** | Draw attention to discrepancies between a person's current behaviour (in terms of the form, frequency, duration, or intensity of that behaviour) and the person's previously set out- come goals, behavioural goals or action plans (goes beyond self-monitoring of behaviour) | Point out that the recorded time spent in SB, or number of prolonged bouts, was more than the goal set of the number of movement breaks was less than the goal set. | Enablement Incentivisation Coercion | L L L |  | Go, T&T | *Gardner 2015 SLR 2 very promising |
| 1.7 | ***Review outcome goal(s)*** | Review outcome goal(s) jointly with the person and consider modifying goal(s) in light of achievement. This may lead to re-setting the same goal, a small change in that goal or setting a new goal instead of, or in addition to the first | Review the outcome (of the behaviour) goal with the person e.g. to be able get up from sitting in < 30 seconds, or TUG time or, VAS pain score (progress / achievement), adapt or choose new goal accordingly. | Enablement | M | Go,  Q1 | Go, T&T | Gardner 2015 1 v promising |
| 1.8 | ***Behavioural contract*** | Create a written specification of the behaviour to be performed, agreed by the person, and witnessed by another | Sign a contract with the person e.g. specifying that they will not sit for > 50 minutes / hour for 1 week, or that they will take a movement break every hour. | Incentivisation Coercion Enablement | L L L | In  Q1 |  |  |
| 1.9 | ***Commitment*** | Ask the person to affirm or reaffirm statements indicating commitment to change the behaviour | Ask the person to use an 'I will' statement to affirm or reaffirm a strong commitment (i.e. using the words 'strongly', 'committed' or 'high priority') to start, continue or restart the attempt to reduce SB / increase sedentary breaks. 'I commit to taking 4 movement breaks during evening TV', 'It is a high priority for me to place my phone out of reach'. | Incentivisation Coercion Enablement | L L L | In  Q1 |  | *Gardner 2015 SLR 2 promising |
| 2.1 | ***Monitoring of behaviour by others without feedback*** | Observe or record behaviour with the person's knowledge as part of a behaviour change strategy | Record the persons pattern of SB using a device which include postural element and time points, of frequency of breaks and bouts, and if possible context. | Incentivisation Coercion | M M |  |  | Gardner 2015 SLR promise ratio 1.0 |
| 2.2 | ***Feedback on behaviour*** | Monitor and provide informative or evaluative feedback on performance of the behaviour *(e.g. form, frequency, duration, intensity)* | Inform the person of the time they spent sedentary in minutes per day, and how many prolonged bouts or how many movement breaks the took. | Education Persuasion Incentivisation Coercion Training | M M M M M | Kn, Q1 & Q2 | Br,  T&T | *Gardner 2015 SLR promise ratio 2.0  Most potent SLR1 Curran et al 2021 |
| 2.3 | ***Self-monitoring of behaviour*** | Establish a method for the person to monitor and record their behaviour(s) as part of a behaviour change strategy | Ask the person to use a daily diary to record when they start and stop sitting, and how many movement breaks they take.  Use a wearable device (accelerometer with postural component) with phone app showing time spent sedentary, sedentary bouts and breaks. | Education Incentivisation Coercion Training Enablement | M M M M M | Br, Q1 & Q2 | Br, T&T | *Gardner 2015 SLR promise ratio 4.0 Curran et al 2021 (with prompts cues and adding objects -promise) Compernolle SLR (if device monitored) PA in PwO Carraca 2021 (SLR BCTs PA PwO)negative effect |
| 2.4 | ***Self-monitoring of outcome(s) of behaviour*** | Establish a method for the person to monitor and record the **outcome(s)** of their behaviour as part of a behaviour change strategy | Ask person to record in diary over 2 weeks, how they feel (emotional / pain / energy) before and after SB movement break e.g. HADS, pain on VAS. | Education Incentivisation Coercion Training Enablement | L L L L L |  |  | Gardner 2015 SLR promise ratio 0.5 |
| 2.5 | ***Monitoring outcome(s) of behaviour by others without feedback*** | Observe or record outcomes of behaviour with the person's knowledge as part of a behaviour change strategy | Agree with person to record their heart rate, BP, QOL, pain / emotional scales, measures of fitness (via app / diary) | Incentivisation Coercion | M M |  |  |  |
| 2.6 | ***Biofeedback*** | Provide feedback about the body (e.g. physiological or biochemical state) using an external monitoring device as part of a behaviour change strategy | Use a wearable device (e.g. accelerometer with inclinometry function) with display of sedentary time and number of movement breaks and heartrate (e.g. Via app or directly on device) | Education Persuasion Incentivisation Coercion Training | L L L L L | Kn, Q1 & Q2 | Kn, T&T | Gardner 2015 1 unpromising |
| 2.7 | ***Feedback on outcome(s) of behaviour*** | Monitor and provide feedback on the outcome of performance of the behaviour | Inform the person of change in heartrate / BP / QOL measures, emotional/pain scales, associated with change in posture / energy expenditure / reduced SB. | Education Persuasion Incentivisation Coercion Training | M M M M M |  |  | Gardner 2015 1 promising |
| 3.1 | ***Social support (unspecified)*** | Advise on, arrange or provide social support (e.g. from friends, relatives, colleagues,' buddies' or staff) or non-contingent praise or reward for performance of the behaviour. It includes encouragement and counselling, but only when it is directed at the **behaviour** | Advise the person to chat to family / housemates when they feel like screentime, Arrange for housemate to encourage movement breaks / non SB | Enablement | M | SI, Q1 | Br, T&T | *Gardner 2015 SLR promise ratio 2.3 |
| 3.2 | ***Social support (practical)*** | Advise on, arrange, or provide **practical** help *(e.g. from friends, relatives, colleagues, ‘buddies’ or staff) for performance of the behaviour* | Ask the persons housemate not to bring refreshment to the person when at screens / when watching T.V or to ask the person to bring refreshment for both (e.g. hot beverage).  Arrange for person to attend online seated movement class, e.g. chair yoga | Enablement | M | SI, Q1 | Br, ECR, T&T | *Gardner 2015 SLR promise ratio 2.0 |
| 3.3 | ***Social support (emotional)*** | Advise on, arrange, or provide **emotional** social support *(e.g. from friends, relatives, colleagues, ‘buddies’ or staff) for performance of the behaviour* | Advise / arrange for person to attend online support for achieving non SB for PwO | Enablement | L | SI, Em, Q1 & Q2 |  |  |
| 4.1 | ***Instruction on how to perform a behaviour*** | Advise or agree on how to perform the behaviour (includes '**Skills training**') | Advise the person how to increase seated energy expenditure / how often to take sedentary breaks. | Training | M |  | Sk, Kn, BaCa, T&T | *Gardner 2015 SLR promise ratio 1.6 Curran et al 2021(some promise) |
| 4.2 | ***Information about antecedents*** | Provide information about antecedents (e.g. social and environmental situations and events, emotions, cognitions) that reliably predict performance of the behaviour | Advise person to keep a record of emotions / activities / people present prior to prolonged SB | Education | L | Kn, Q1 & Q2 | Kn, Br, T&T |  |
| 4.3 | ***Re-attribution*** | Elicit perceived causes of behaviour and suggest alternative explanations (e.g. external or internal and stable or unstable). | If the person attributes their SB to the frequent presence of pain suggest that the 'real' cause may be the persons inattention to taking pain medication and pacing activity. | Education Persuasion | L L |  |  |  |
| 4.4 | ***Behavioural experiments*** | Advise on how to identify and test hypotheses about the behaviour, its causes and consequences, by collecting and interpreting data | Ask person to keep record of physical and emotional feeling after taking SB breaks / movement breaks | Education Persuasion Training Enablement | L L L L |  |  |  |
| 5.1 | ***Information about health consequences*** | Provide information (e.g. written, verbal, visual) about health consequences of performing the behaviour | Explain that non SB maintains / improves mobility / cardiovascular health; SB increases susceptibility to CVD. | Education Persuasion | M M | Kn, Q1 & Q2 | Kn, BaCo, In,  T&T | *Gardner 2015 SLR promise ratio 2.7 Curran et al 2021(some promise) |
| 5.2 | ***Salience of consequences*** | Use methods specifically designed to emphasise the consequences of performing the behaviour with the aim of making them more memorable (goes beyond informing about consequences) | Produce adverts showing pictures of health consequences e.g. graphic depiction of heart disease / stroke, person imprisoned at home due to immobility, resulting from SB. Produce adverts of PwO looking and feeling happy and healthy, engaged, in non-sedentary activity / movement /social situation. | Persuasion Enablement | L L | BaCo, Q1 | BaCo, T&T |  |
| 5.3 | ***Information about social and environmental consequences*** | Provide information (e.g. written, verbal, visual) about social and environmental consequences of performing the behaviour | Inform person about benefit of engaging in non-sedentary activity / movement with children grandchildren / pets / friends (social benefit). | Education Persuasion | M M | BaCo, Q1 | Kn, BaCo, T&T | Gardner 2015 SLR promise ratio 1.0 |
| 5.4 | ***Monitoring of emotional consequences*** | Prompt assessment of **feelings** after attempts at performing the behaviour | Agree that the person will record how they feel after taking their sedentary breaks / movement break | Enablement | L | Em, Q1 & Q2 |  |  |
| 5.5 | ***Anticipated regret*** | Induce or raise awareness of expectations of future regret about performance of the unwanted behaviour | Ask the person to assess the degree of regret they will feel if they do not take a movement break / increase seated energy expenditure | Coercion Enablement | L L | BaCo, Q1 | BaCo, T&T |  |
| 5.6 | ***Information about emotional consequences*** | Provide information (e.g. written, verbal, visual) about emotional consequences of performing the behaviour | Explain ( e.g. verbally, provide leaflets, by video) that SB is linked to anxiety / depression and non SB is related to reduced anxiety / depression/ happiness | Education Persuasion | L L | BaCo, Em, Q1 & Q2 | BaCo, T&T |  |
| 6.1 | ***Demonstration of the behaviour*** | Provide an observable sample of the performance of the behaviour, directly in person or indirectly e.g. via film, pictures, for the person to aspire to or imitate (includes 'Modelling') | Demonstrate to the person how to increase energy expenditure in sitting e.g. weights, bands, pedalling, chair activities. | Training Modelling | M M | SI,  Q1 | BaCa, T&T |  |
| 6.2 | ***Social comparison*** | Draw attention to others' performance to allow comparison with the person's own performance | Identify and show other PwO with similar abilities engaged in non-sedentary activity, e.g. gardening, getting up to let dog out and choosing to do some activity. | Persuasion | L | SI,  Q1 |  | *Gardner 2015 SLR 2 promising |
| 6.3 | ***Information about others’ approval*** | Provide information about what other people think about the behaviour. The information clarifies whether others will like, approve or disapprove of what the person is doing or will do | Tell the person that other PwO approve of meeting non sedentary guidelines, children / family like to see person moving. | Persuasion Education | L L | SI,  Q1 |  |  |
| 7.1 | ***Prompts/ cues*** | Introduce or define environmental or social stimulus with the purpose of prompting or cueing the behaviour. The prompt or cue would normally occur at the time or place of performance. | Put a sticker on the remote control to remind person to delay TV time / take a movement break when changing channel. Put an auditory sensor mat in front of sofa or sitting room to remind person to delay sitting | Education Environment restructure | M M | ECR,  Q1 | MADP,  ECR, T&T | *Gardner 2015 SLR promise ratio 2.0 Curran et al 2021 (freq used with adding objects and self-monitoring) |
| 7.2 | ***Cue signalling reward*** | Identify an environmental stimulus that reliably predicts that reward will follow the behaviour (includes '**Discriminative cue**') | Advise the person to lock the remote control out of reach of the sitting position, so that standing up is rewarded by change of channel. | Education Environment restructure Incentivisation | L L L | ECR, Q1 |  |  |
| 7.3 | ***Reduce prompts/ cues*** | Withdraw gradually prompts to perform the behaviour (includes '**Fading**') | Reduce gradually the number of reminders used to prompt standing | Environment restructure | L |  |  |  |
| 7.4 | ***Remove access to the reward*** | Advise or arrange for the person to be separated from situations in which unwanted behaviour can be rewarded in order to reduce the behaviour (includes '**Time out**') | To remove access to screen time, set timers to switch off WIFI / TV. or  Advise person to place all screens in a locked room / Lock TV room. | Coercion Environment restructure | L L |  |  |  |
| 7.5 | ***Remove aversive stimulus*** | Advise or arrange for the removal of an aversive stimulus to facilitate behaviour change (includes '**Escape learning**') | Arrange for family to stop nagging the person to get off the sofa in order to increase non SB.  Advise person to visit doctor re pain relief | Incentivisation Environment restructure | L L |  | ECR, T&T |  |
| 7.6 | ***Satiation*** | Advise or arrange repeated exposure to a stimulus that reduces or extinguishes a drive for the unwanted behaviour | Arrange for the person to watch TV / screens without breaks for a full day / 2 days until the desire to do so diminishes | Education Environment restructure | L L |  |  |  |
| 7.7 | ***Exposure*** | Provide systematic confrontation with a feared stimulus to reduce the response to a later encounter | Agree a schedule with person who believes pain will increase with standing / moving, to gradually spend time in standing / moving. | Environment restructure | L |  |  |  |
| 7.8 | ***Associative learning*** | Present a neutral stimulus jointly, with a stimulus that already elicits the behaviour, repeatedly until the neutral stimulus elicits that behaviour (includes '**Classical/Pavlovian Conditioning**') | To get a person to stand when using social media, place a sticker on bathroom mirror (using comfort break as neutral stimulus) to remind them to check social media in standing before sitting back at TV | Environment restructure | L | Re, |  |  |
| 8.1 | ***Behavioural practice/ rehearsal*** | Prompt practice or rehearsal of the performance of the behaviour one or more times in a context or at a time when the performance may not be necessary, in order to increase habit and skill | Prompt person to practice seated movements | Training | M | Sk, Q1 & Q2 | Sk, BaCa, T&T | *Gardner 2015 SLR >2 promising  PA in PwO (Carraca 2021) mediator |
| 8.2 | ***Behaviour substitution*** | Prompt substitution of the unwanted behaviour with a wanted or neutral behaviour *Note: if this occurs regularly, also code* ***8.4, Habit reversal*** | Suggest that the person walks around the garden rather than watches TV Suggest that the person stand during online activity / replace with active online activity. | Enablement | L |  | Br, T&T | *Gardner 2015 SLR promise ratio 2.5 |
| 8.3 | ***Habit formation*** | Prompt rehearsal and repetition of the behaviour in the same context repeatedly so that the context elicits the behaviour Note: also code ***8.1, Behavioural practice/rehearsal*** | Ask the person to use pedals / resistance bands / weights every time they watch favourite TV programme. | Training | L | Sk, Q1 & Q2 |  | *Gardner 2015 SLR >2 promising |
| 8.4 | ***Habit reversal*** | Prompt rehearsal and repetition of an alternative behaviour to **replace** an unwanted habitual behaviour | Ask the person to make own beverage during screen time rather than have family / housemate bring it. Ask person to stand intermittently during online activity. | Training | L | Sk, Q1 & Q2 |  | Gardner 2015 1 promising |
| 8.5 | ***Overcorrection*** | Ask to repeat the wanted behaviour in an exaggerated way following an unwanted behaviour | Ask to limit sitting to 5 minutes next half hour, if sat for > 1 hour. | Enablement | L |  |  |  |
| 8.6 | ***Generalisation of a target behaviour*** | Advise to perform the wanted behaviour, which is already performed in a particular situation, in another situation | Advise to repeat exercises learned in the gym / WMC when at home. | Enablement | L |  |  |  |
| 8.7 | ***Graded tasks*** | Set easy-to-perform tasks, making them increasingly difficult, but achievable, until behaviour is performed | Ask the person to perform seated exercise (or stand) for 2 minute every other hour; then 2 minutes per hour; then 5 minutes per hour, then 5 minutes / half hour. Progressing only when prior target achieved. | Training Enablement | L L | Sk, Q1 & Q2 | Sk,  BaCa, T&T | *Gardner 2015 SLR promise ratio 1.2 |
| 9.1 | ***Credible source*** | Present verbal or visual communication from a **credible source** in favour of or against the behaviour | Present a speech given by a PwO or obesity specialist / HCP to emphasise the importance of non SB for PwO. | Persuasion | M | BaCo, Q1 & Q2 |  | Gardner 2015 SLR promise ratio 1.0 |
| 9.2 | ***Pros and cons*** | Advise the person to identify and compare reasons for wanting (pros) and not wanting to (cons) change the behaviour (includes '**Decisional balance**') | Advise the person to list and compare the advantages of reducing SB / vs prolonged SB. | Enablement | L | BaCo, Q1 | BaCo T&T | *Gardner 2015 SLR >2 promising |
| 9.3 | ***Comparative imagining of future outcomes*** | Prompt or advise the imagining and comparing of future outcomes of changed versus unchanged behaviour | Prompt the person to imagine and compare likely or possible outcomes of prolonged SB vs interrupting / minimising SB | Enablement | L | BaCo, Q1 | BaCo, T&T |  |
| 10.1 | ***Material incentive (behaviour)*** | Inform that money, vouchers or other valued objects will be delivered if and only if there has been effort and/or progress in performing the behaviour (includes 'Positive reinforcement') | Inform that a financial payment will be each day meeting SB guidelines | Incentivisation | L | Re, | BaCo, Re, T&T | Gardner 2015 SLR 1 v promising |
| 10.2 | ***Material reward (behaviour)*** | Arrange for the delivery of money, vouchers or other valued objects if and only if there has been effort and/or progress in performing the behaviour (includes **‘Positive reinforcement’**) | Arrange for the person to receive a voucher if daily number of movement breaks increases or daily time spent sedentary decreases consistently or meets targets over a month. | Incentivisation | L | Re, | Re, | Gardner 2015 SLR 1 v promising |
| 10.3 | ***Non-specific reward*** | Arrange delivery of a reward if and only if there has been effort and/or progress in performing the behaviour (includes '**Positive reinforcement**') | Identify something (e.g. an activity such as a visit to an art museum) that the person values and arrange for this to be delivered if and only if they reduce SB | Incentivisation | L | Re, | Re, |  |
| 10.4 | ***Social reward*** | Arrange verbal or non-verbal reward if and only if there **has been** effort and/or progress in performing the behaviour (includes 'Positive reinforcement') | Congratulate the person for each day they reduce SB / take more movement breaks | Incentivisation | L | SI, Re, Q1 | Re, | *Gardner 2015 SLR >2 promising |
| 10.5 | ***Social incentive*** | Inform that a verbal or non-verbal reward will be delivered if and only if there has been effort and/or progress in performing the behaviour (includes '**Positive reinforcement**') | Inform person that they will be congratulated for each day they meet non SB target | Incentivisation | N |  |  |  |
| 10.6 | ***Non-specific incentive*** | Inform that a reward will be delivered if and only if there has been effort and/or progress in performing the behaviour (includes '**Positive reinforcement**') | Identify an activity that the person values and inform them that this will happen if and only if they take hourly SB breaks | Incentivisation | L | Re, | Re, |  |
| 10.7 | ***Self-incentive*** | Plan to reward self in future if and only if there has been effort and/or progress in performing the behaviour | Encourage to reward self with material (e.g., new clothes) or other valued objects if and only if they have adhered to a non-SB targets | Incentivisation | L |  |  |  |
| 10.8 | ***Incentive (outcome)*** | Inform that a reward ***will be*** delivered if and only if there has been effort and/or progress in achieving the behavioural **outcome** (*includes* '**Positive reinforcement**') | Inform the person that they will receive money if and only if non SB targets are met | Incentivisation | N |  | In, BaCo, Re, T&T |  |
| 10.9 | ***Self-reward*** | Prompt self-praise or self-reward if and only if there ***has been*** effort and/or progress in performing the behaviour | Encourage the person to reward self with material (e.g., new clothes / personal care treatment) or other valued objects if and only if they have adhered to a non-SB targets | Incentivisation Training Enablement | L L L | Re, |  | Gardner 2015 SLR 1 promising |
| 10.10. | ***Reward(outcome)*** | Arrange for the delivery of a reward if and only if there ***has been*** effort and/or progress in achieving the behavioural outcome (includes 'Positive reinforcement') | Arrange for the person to receive money if and only if non SB targets are met | Incentivisation | L |  | BaCo, In, T&T |  |
| 10.1 | ***Future punishment*** | Inform that future punishment or removal of reward will be a consequence of performance of an unwanted behaviour (may include fear arousal) (includes '**Threat**') | Inform that prolonged SB is likely to result in loss of mobility and increased pain if the person continues | Coercion | L |  |  |  |
| 11.1 | ***Pharmacological support*** | Provide, or encourage the use of or adherence to, drugs to facilitate behaviour change | Suggest the patient asks GP for appropriate medications for pain / mental health / obesity / other limiting co-morbidity | Enablement | L | Re, |  |  |
| 11.2 | ***Reduce negative emotions*** | Advise on ways of reducing negative emotions to facilitate performance of the behaviour (includes '**Stress Management**') | Advise on the use of stress management skills, e.g. breathing exercises and mindful movement to reduce feeling incapable of non SB or that it is worthless, or internal bias. | Enablement | L | Em, Q1 & Q2 | MADP, Em, Br, T&T |  |
| 11.3 | ***Conserving mental resources*** | Advise on ways of minimising demands on mental resources to facilitate behaviour change | Advise on using reminders to raise awareness of prolonged sitting. | Enablement | L |  | Br, MADP, T&T |  |
| 11.4 | ***Paradoxical instructions*** | Advise to engage in some form of the unwanted behaviour with the aim of reducing the motivation to engage in that behaviour. | Advise the person to watch TV / screens without breaks for a full day / 2 days until the desire to do so diminishes | Incentivisation | L |  |  |  |
| 12.1 | ***Restructuring the physical environment*** | Change, or advise to change the physical environment in order to facilitate performance of the wanted behaviour or create barriers to the unwanted behaviour (other than prompts/ cues, rewards and punishments) | Advise to keep screens / ipads/ phone / remote control in an inconvenient place. Advise person to use / change seating too easy to stand from seats. | Enablement Environment restructure | M M | ECR,  Q1 | ECR,  T&T | *Gardner 2015 SLR >2 v promising |
| 12.2 | ***Restructuring the social environment*** | Change, or advise to change the social environment in order to facilitate performance of the wanted behaviour or create barriers to the unwanted behaviour (other than prompts/ cues, rewards and punishments) | Encourage person to engage in online movement activity. Advise the person to invite friends / family who are active to their home / suggest active engagement with family / friends (e.g. active gaming) | Enablement Environment restructure | L L | ECR, SI, Q1 & Q2 | ECR,  T&T | *Gardner 2015 SLR promise ratio 3.0 |
| 12.3 | ***Avoidance/ reducing exposure to cues for the behaviour*** | Advise on how to avoid exposure to specific social and contextual/physical cues for the behaviour, including changing daily or weekly routines | Suggest to a person who wants to reduce leisure screen time that screens / devices are placed out of reach, in one specific place (not in usual room) | Enablement Environment restructure | L N | ECR,  Q1 & Q2 | ECR,  T&T |  |
| 12.4 | ***Distraction*** | Advise or arrange to use an alternative focus for attention to avoid triggers for unwanted behaviour | Suggest to a person who is trying to delay sitting, to focus on a topic they enjoy (e.g. going to the garden) | Enablement | L |  |  |  |
| 12.5 | ***Adding objects to the environment*** | Add objects to the environment in order to facilitate performance of the behaviour | Provide pedals / resistance bands / pedometer, mobility aids. | Enablement Environment restructure | M M |  | ECR,  T&T | *Gardner 2015 SLR promise ratio 1.7 Curran et al 2021 (small wearables also using prompts and cues; self-monitoring ) Shresta 2017 Movement objects e.g. pedals effective |
| 12.6 | ***Body changes*** | Alter body structure, functioning or support **directly** to facilitate behaviour change | Prompt strength training, balance training or provide assistive aids (e.g. mobility aid, aids for seated activity) | Enablement | L | Sk  Q1 |  |  |
| 13.1 | ***Identification of self as role model*** | Inform that one's own behaviour may be an example to others | Inform the person that if they take frequent breaks from screens they may be a good example for their children | Persuasion Enablement | L L | SI,  Q1 |  | Gardner 2015 SLR 1 promising |
| 13.2 | ***Framing/ reframing*** | Suggest the deliberate adoption of a perspective or new perspective on behaviour (e.g. its purpose) in order to change cognitions or emotions about performing the behaviour (includes '**Cognitive structuring**'). | Suggest that the person might think of the tasks as maintaining mobility and ability rather than reducing sedentary behaviour (or losing weight / increasing PA) | Persuasion Enablement | L L |  |  | Ramos Salas (2019) counter narratives may be important for obesity bias / stigma & its effect on movement Q1 & Q2 |
| 13.3 | ***Incompatible beliefs*** | Draw attention to discrepancies between current or past behaviour and self-image, in order to create discomfort (includes 'Cognitive dissonance') | Draw attention to a person’s SB at home but also high level of PA / Identity as active person; | Coercion Enablement | L L |  |  |  |
| 13.4 | ***Valued self-identity*** | Advise the person to write or complete rating scales about a cherished value or personal strength as a means of affirming the person's identity as part of a behaviour change strategy (includes 'Self-affirmation') | Advise the person to write about their personal strengths before they receive a message advocating for movement breaks, reduced SB | Enablement | L |  |  |  |
| 13.5 | ***Identity associated with changed behaviour*** | Advise the person to construct a new self-identity as someone who 'used to engage with the unwanted behaviour' | Ask the person to articulate their new identity as a 'non sedentary person' or person who 'used to be sedentary' | Persuasion Enablement | L L |  |  |  |
| 14.1 | ***Behaviour cost*** | Arrange for withdrawal of something valued if and only if an unwanted behaviour is performed (includes 'Response cost'). Note if withdrawal of contingent reward code, 14.3, Remove reward | Subtract money from a prepaid refundable deposit when sitting / reclining for >1hour | Coercion | L | Re, |  |  |
| 14.2 | ***Punishment*** | Arrange for aversive consequence contingent on the performance of the unwanted behaviour | Arrange for the person to wear unattractive clothes following prolonged SB | Coercion | L | Re, | Re, |  |
| 14.3 | ***Remove reward*** | Arrange for discontinuation of contingent reward following performance of the unwanted behaviour(includes extinction) | Arrange for the other people in the household to ignore the person every time they sit / recline for prolonged time (rather than attending to them by criticising or persuading) | Coercion | L | Re, |  |  |
| 14.4 | ***Reward approximation*** | Arrange for reward following any approximation to the target behaviour, gradually rewarding only performance closer to the wanted behaviour (includes '**Shaping**') | Arrange reward for any decrease in SB, gradually requiring the daily non SB to become closer to the planned reduction in SB | Incentivisation | L | Re, |  |  |
| 14.5 | ***Rewarding completion*** | Build up behaviour by arranging reward following final component of the behaviour; gradually add the components of the behaviour that occur earlier in the behavioural sequence (includes '**Backward chaining**') | Reward standing from sitting; then make reward contingent on moving to get the reward; then make re- ward contingent on moving for 5 minutes. | Incentivisation | L |  |  |  |
| 14.6 | ***Situation specific reward*** | Arrange for reward following the behaviour in one situation but not in another (includes '**Discrimination training**') | Arrange reward for moving during TV time but not during mealtime | Incentivisation | L | Re, |  |  |
| 14.7 | ***Reward incompatible behaviour*** | Arrange reward for responding in a manner that is incompatible with a previous response to that situation (includes '**Counter-conditioning**') | Arrange reward for using standing desk for social screentime | Incentivisation | L | Re, |  |  |
| 14.8 | ***Reward alternative behaviour*** | Arrange reward for performance of an alternative to the unwanted behaviour (includes '**Differential reinforcement**') | Reward for LPA but not for SB | Incentivisation | L | Re, |  |  |
| 14.9 | ***Reduce reward frequency*** | Arrange for rewards to be made contingent on increasing duration or frequency of the behaviour (includes 'Thinning') | Arrange reward for each day the person takes hourly SB breaks, then each week, then each month, then every 2 months and so on | Incentivisation | L | Re, |  |  |
| 14.10. | ***Remove punishment*** | Arrange for removal of an unpleasant consequence contingent on performance of the wanted behaviour (includes '**Negative reinforcement**') | Arrange for necessary home repairs (e.g. painting) only if non-SB / movement breaks consistently performed for a week. | Incentivisation | L | Re, |  |  |
| 15.1 | ***Verbal persuasion about capability*** | Tell the person that they can successfully perform the wanted behaviour, arguing against self-doubts and asserting that they can and will succeed | Tell the person that they can successfully decrease their SB, despite their pain and bodyweight. | Persuasion Enablement | L L | Op BaCa, Q1 & Q2 | BaCa, T&T | Gardner 2015 1 unpromising |
| 15.2 | ***Mental rehearsal of successful performance*** | Advise to practice imagining performing the behaviour successfully in relevant contexts | Advise the person to imagine easily getting up from sitting / performing movement break. | Training  Enablement | L L |  |  |  |
| 15.3 | ***Focus on past success*** | Advise to think about or list previous successes in performing the behaviour (or parts of it) | Advise the person to describe or list previous times when SB was replaced with non-SB. | Persuasion Enablement | L L | BaCa, Q1 & Q2 | BaCa, T&T |  |
| 15.4 | ***Self-talk*** | Prompt positive self-talk (aloud or silently) before and during the behaviour | Prompt the person to tell themselves that all movement matters and / that or a movement break will be energising, will be worth it, is maintaining mobility, enhancing health. | Training  Enablement | L L |  | BaCa, T&T | although no BCTs mentioned in (Ramos Salas 2019) counter narratives for obesity bias and stigma and its effect on movement seem important from Q1 & Q2 |
| 16.1 | ***Imaginary punishment*** | Advise to imagine performing the **unwanted** behaviour in a real-life situation followed by imagining an unpleasant consequence (includes 'Covert sensitisation') | Advise person to imagine feeling depressed or in pain after prolonged SB | Coercion Enablement | L L |  |  |  |
| 16.2 | ***Imaginary reward*** | Advise to imagine performing the **wanted** behaviour in a real-life situation followed by imagining a pleasant consequence (includes 'Covert conditioning') | Advise the person to imagine taking a movement break from screen followed by feeling pain free and mobile. | Incentivisation Enablement | L L | BaCo, |  |  |
| 16.3 | ***Vicarious consequences*** | Prompt observation of the consequences (including rewards and punishments) for others when they perform the behaviour | Prompt person to observe a PwO in a movement break feeling pain-free and happy;  Draw attention to the positive comments other PwO get when they reduce SB | Enablement | L | BaCo, SI, Q1 |  |  |

**Table S8.** Longlist BCT’s with potential to change SB

| BCT No. | BCT Label | Definition | Example For SB reduction | Intervention functions targeted |
| --- | --- | --- | --- | --- |
| 1.1 | ***Goal setting (behaviour)*** | Set or agree a goal defined in terms of the behaviour to be achieved | Agree a daily maximum sitting time or number of movement breaks per hour | Enablement |
| 1.2 | ***Problem solving*** | Analyse , or prompt the person to analyse, factors influencing the behaviour and generate or select strategies that include overcoming barriers and/or increasing facilitators (includes ***‘Relapse Prevention’ and ‘Coping Planning’***) | Identify specific triggers (e.g. feeling low/ anxious/tired /pain) that initiate and sustain prolonged sedentary / sitting time and develop strategies for avoiding environmental triggers or for managing e.g. negative emotions, such as anxiety, or physical pain that motivate SB | Enablement |
| 1.3 | ***Goal setting (outcome)*** | Set or agree a goal defined in terms of a positive **outcome** of wanted behaviour | Set a time score e.g. to be able get up from sitting in < 30 seconds, or TUG time or, VAS pain score, or sit stand chair test. | Enablement |
| 1.4 | ***Action planning*** | Prompt detailed planning of performance of the behaviour (must include at least one of context, frequency, duration and intensity). Context may be environmental (physical or social) or internal (physical, emotional or cognitive) (includes ***‘Implementation Intentions’***) | Prompt planning taking a movement breaks from evening TV, identifying ad breaks as cues to move, allowing one break to be ignored, move every second break when feeling tired / stressed. | Enablement |
| 1.5 | ***Review behaviour goal(s)*** | Review behaviour goal(s) jointly with the person and consider modifying goal(s) or behaviour change strategy in light of achievement. This may lead to re-setting the same goal, a small change in that goal or setting a new goal instead of (or in addition to) the first, or no change | Examine how well a person's performance corresponds to agreed goals e.g. whether they took SB breaks hourly, and consider modifying future behavioural goals accordingly e.g. by increasing or decreasing SB break targets | Enablement |
| 1.6 | ***Discrepancy between current behaviour and goal*** | Draw attention to discrepancies between a person's current behaviour (in terms of the form, frequency, duration, or intensity of that behaviour) and the person's previously set out- come goals, behavioural goals or action plans (goes beyond self-monitoring of behaviour) | Point out that the recorded time spent in SB, or number of prolonged bouts, was more than the goal set of the number of movement breaks was less than the goal set. | Enablement Incentivisation Coercion |
| 1.7 | ***Review outcome goal(s)*** | Review outcome goal(s) jointly with the person and consider modifying goal(s) in light of achievement. This may lead to re-setting the same goal, a small change in that goal or setting a new goal instead of, or in addition to the first | Review the outcome (of the behaviour) goal with the person e.g. to be able get up from sitting in < 30 seconds, or TUG time or, VAS pain score (progress / achievement), adapt or choose new goal accordingly. | Enablement |
| 1.9 | ***Commitment*** | Ask the person to affirm or reaffirm statements indicating commitment to change the behaviour | Ask the person to use an 'I will' statement to affirm or reaffirm a strong commitment (i.e. using the words 'strongly', 'committed' or 'high priority') to start, continue or restart the attempt to reduce SB / increase sedentary breaks. 'I commit to taking 4 movement breaks during evening TV', 'It is a high priority for me to place my phone out of reach'. | Incentivisation Coercion Enablement |
| 2.2 | ***Feedback on behaviour*** | Monitor and provide informative or evaluative feedback on performance of the behaviour *(e.g. form, frequency, duration, intensity)* | Inform the person of the time they spent sedentary in minutes per day, and how many prolonged bouts or how many movement breaks the took. | Education Persuasion Incentivisation Coercion Training |
| 2.3 | ***Self-monitoring of behaviour*** | Establish a method for the person to monitor and record their behaviour(s) as part of a behaviour change strategy | Ask the person to use a daily diary to record when they start and stop sitting, and how many movement breaks they take.  Use a wearable device (accelerometer with postural component) with phone app showing time spent sedentary, sedentary bouts and breaks. | Education Incentivisation Coercion Training Enablement |
| 2.6 | ***Biofeedback*** | Provide feedback about the body (e.g. physiological or biochemical state) using an external monitoring device as part of a behaviour change strategy | Use a wearable device (e.g. accelerometer with inclinometry function) with display of sedentary time and number of movement breaks and heartrate (e.g. Via app or directly on device) | Education Persuasion Incentivisation Coercion Training |
| 2.7 | ***Feedback on outcome(s) of behaviour*** | Monitor and provide feedback on the outcome of performance of the behaviour | Inform the person of change in heartrate / BP / QOL measures, emotional/pain scales, associated with change in posture / energy expenditure / reduced SB. | Education Persuasion Incentivisation Coercion Training |
| 3.1 | ***Social support (unspecified)*** | Advise on, arrange or provide social support (e.g. from friends, relatives, colleagues,' buddies' or staff) or non-contingent praise or reward for performance of the behaviour. It includes encouragement and counselling, but only when it is directed at the **behaviour** | Advise the person to chat to family / housemates when they feel like screentime, Arrange for housemate to encourage movement breaks / non SB | Enablement |
| 3.2 | ***Social support (practical)*** | Advise on, arrange, or provide **practical** help *(e.g. from friends, relatives, colleagues, ‘buddies’ or staff) for performance of the behaviour* | Ask the persons housemate not to bring refreshment to the person when at screens / when watching T.V or to ask the person to bring refreshment for both (e.g. hot beverage).  Arrange for person to attend online seated movement class, e.g. chair yoga | Enablement |
| 3.3 | ***Social support (emotional)*** | Advise on, arrange, or provide **emotional** social support *(e.g. from friends, relatives, colleagues, ‘buddies’ or staff) for performance of the behaviour* | Advise / arrange for person to attend online support for achieving non SB for PwO | Enablement |
| 4.1 | ***Instruction on how to perform a behaviour*** | Advise or agree on how to perform the behaviour (includes '**Skills training**') | Advise the person how to increase seated energy expenditure / how often to take sedentary breaks. | Training |
| 4.2 | ***Information about antecedents*** | Provide information about antecedents (e.g. social and environmental situations and events, emotions, cognitions) that reliably predict performance of the behaviour | Advise person to keep a record of emotions / activities / people present prior to prolonged SB | Education |
| 5.1 | ***Information about health consequences*** | Provide information (e.g. written, verbal, visual) about health consequences of performing the behaviour | Explain that non SB maintains / improves mobility / cardiovascular health; SB increases susceptibility to CVD. | Education Persuasion |
| 5.2 | ***Salience of consequences*** | Use methods specifically designed to emphasise the consequences of performing the behaviour with the aim of making them more memorable (goes beyond informing about consequences) | Produce adverts showing pictures of health consequences e.g. graphic depiction of heart disease / stroke, person imprisoned at home due to immobility, resulting from SB. Produce adverts of PwO looking and feeling happy and healthy, engaged, in non-sedentary activity / movement /social situation. | Persuasion Enablement |
| 5.3 | ***Information about social and environmental consequences*** | Provide information (e.g. written, verbal, visual) about social and environmental consequences of performing the behaviour | Inform person about benefit of engaging in non-sedentary activity / movement with children grandchildren / pets / friends (social benefit). | Education Persuasion |
| 5.4 | ***Monitoring of emotional consequences*** | Prompt assessment of **feelings** after attempts at performing the behaviour | Agree that the person will record how they feel after taking their sedentary breaks / movement break | Enablement |
| 5.6 | ***Information about emotional consequences*** | Provide information (e.g. written, verbal, visual) about emotional consequences of performing the behaviour | Explain ( e.g. verbally, provide leaflets, by video) that SB is linked to anxiety / depression and non SB is related to reduced anxiety / depression/ happiness | Education Persuasion |
| 6.1 | ***Demonstration of the behaviour*** | Provide an observable sample of the performance of the behaviour, directly in person or indirectly e.g. via film, pictures, for the person to aspire to or imitate (includes 'Modelling') | Demonstrate to the person how to increase energy expenditure in sitting e.g. weights, bands, pedalling, chair activities. | Training Modelling |
| 6.2 | ***Social comparison*** | Draw attention to others' performance to allow comparison with the person's own performance | Identify and show other PwO with similar abilities engaged in non-sedentary activity, e.g. gardening, getting up to let dog out and choosing to do some activity. | Persuasion |
| 7.1 | ***Prompts/ cues*** | Introduce or define environmental or social stimulus with the purpose of prompting or cueing the behaviour. The prompt or cue would normally occur at the time or place of performance. | Put a sticker on the remote control to remind person to delay TV time / take a movement break when changing channel. Put an auditory sensor mat in front of sofa or sitting room to remind person to delay sitting | Education Environment restructure |
| 7.5 | ***Remove aversive stimulus*** | Advise or arrange for the removal of an aversive stimulus to facilitate behaviour change (includes '**Escape learning**') | Arrange for family to stop nagging the person to get off the sofa in order to increase non SB.  Advise person to visit doctor re pain relief | Incentivisation Environment restructure |
| 8.1 | ***Behavioural practice/ rehearsal*** | Prompt practice or rehearsal of the performance of the behaviour one or more times in a context or at a time when the performance may not be necessary, in order to increase habit and skill | Prompt person to practice seated movements | Training |
| 8.2 | ***Behaviour substitution*** | Prompt substitution of the unwanted behaviour with a wanted or neutral behaviour *Note: if this occurs regularly, also code* ***8.4, Habit reversal*** | Suggest that the person walks around the garden rather than watches TV Suggest that the person stand during online activity / replace with active online activity. | Enablement |
| 8.3 | ***Habit formation*** | Prompt rehearsal and repetition of the behaviour in the same context repeatedly so that the context elicits the behaviour Note: also code ***8.1, Behavioural practice/rehearsal*** | Ask the person to use pedals / resistance bands / weights every time they watch favourite TV programme. | Training |
| 8.4 | ***Habit reversal*** | Prompt rehearsal and repetition of an alternative behaviour to **replace** an unwanted habitual behaviour | Ask the person to make own beverage during screen time rather than have family / housemate bring it. Ask person to stand intermittently during online activity. | Training |
| 8.7 | ***Graded tasks*** | Set easy-to-perform tasks, making them increasingly difficult, but achievable, until behaviour is performed | Ask the person to perform seated exercise (or stand) for 2 minute every other hour; then 2 minutes per hour; then 5 minutes per hour, then 5 minutes / half hour. Progressing only when prior target achieved. | Training Enablement |
| 9.1 | ***Credible source*** | Present verbal or visual communication from a **credible source** in favour of or against the behaviour | Present a speech given by a PwO or obesity specialist / HCP to emphasise the importance of non SB for PwO. | Persuasion |
| 9.2 | ***Pros and cons*** | Advise the person to identify and compare reasons for wanting (pros) and not wanting to (cons) change the behaviour (includes '**Decisional balance**') | Advise the person to list and compare the advantages of reducing SB / vs prolonged SB. | Enablement |
| 9.3 | ***Comparative imagining of future outcomes*** | Prompt or advise the imagining and comparing of future outcomes of changed versus unchanged behaviour | Prompt the person to imagine and compare likely or possible outcomes of prolonged SB vs interrupting / minimising SB | Enablement |
| 10.1 | ***Material incentive (behaviour)*** | Inform that money, vouchers or other valued objects will be delivered if and only if there has been effort and/or progress in performing the behaviour (includes 'Positive reinforcement') | Inform that a financial payment will be each day meeting SB guidelines | Incentivisation |
| 10.4 | ***Social reward*** | Arrange verbal or non-verbal reward if and only if there **has been** effort and/or progress in performing the behaviour (includes 'Positive reinforcement') | Congratulate the person for each day they reduce SB / take more movement breaks | Incentivisation |
| 11.2 | ***Reduce negative emotions*** | Advise on ways of reducing negative emotions to facilitate performance of the behaviour (includes '**Stress Management**') | Advise on the use of stress management skills, e.g. breathing exercises and mindful movement to reduce feeling incapable of non SB or that it is worthless, or internal bias. | Enablement |
| 11.3 | ***Conserving mental resources*** | Advise on ways of minimising demands on mental resources to facilitate behaviour change | Advise on using reminders to raise awareness of prolonged sitting. | Enablement |
| 12.1 | ***Restructuring the physical environment*** | Change, or advise to change the physical environment in order to facilitate performance of the wanted behaviour or create barriers to the unwanted behaviour (other than prompts/ cues, rewards and punishments) | Advise to keep screens / ipads/ phone / remote control in an inconvenient place. Advise person to use / change seating too easy to stand from seats. | Enablement Environment restructure |
| 12.2 | ***Restructuring the social environment*** | Change, or advise to change the social environment in order to facilitate performance of the wanted behaviour or create barriers to the unwanted behaviour (other than prompts/ cues, rewards and punishments) | Encourage person to engage in online movement activity. Advise the person to invite friends / family who are active to their home / suggest active engagement with family / friends (e.g. active gaming) | Enablement Environment restructure |
| 12.3 | ***Avoidance/ reducing exposure to cues for the behaviour*** | Advise on how to avoid exposure to specific social and contextual/physical cues for the behaviour, including changing daily or weekly routines | Suggest to a person who wants to reduce leisure screen time that screens / devices are placed out of reach, in one specific place (not in usual room) | Enablement Environment restructure |
| 12.5 | ***Adding objects to the environment*** | Add objects to the environment in order to facilitate performance of the behaviour | Provide pedals / resistance bands / pedometer, mobility aids. | Enablement Environment restructure |
| 12.6 | ***Body changes*** | Alter body structure, functioning or support **directly** to facilitate behaviour change | Prompt strength training, balance training or provide assistive aids (e.g. mobility aid, aids for seated activity) | Enablement |
| 13.1 | ***Identification of self as role model*** | Inform that one's own behaviour may be an example to others | Inform the person that if they take frequent breaks from screens they may be a good example for their children | Persuasion Enablement |
| 13.2 | ***Framing/ reframing*** | Suggest the deliberate adoption of a perspective or new perspective on behaviour (e.g. its purpose) in order to change cognitions or emotions about performing the behaviour (includes '**Cognitive structuring**'). | Suggest that the person might think of the tasks as maintaining mobility and ability rather than reducing sedentary behaviour (or losing weight / increasing PA) | Persuasion Enablement |
| 13.4 | ***Valued self-identity*** | Advise the person to write or complete rating scales about a cherished value or personal strength as a means of affirming the person's identity as part of a behaviour change strategy (includes 'Self-affirmation') | Advise the person to write about their personal strengths before they receive a message advocating for movement breaks, reduced SB | Enablement |
| 15.1 | ***Verbal persuasion about capability*** | Tell the person that they can successfully perform the wanted behaviour, arguing against self-doubts and asserting that they can and will succeed | Tell the person that they can successfully decrease their SB, despite their pain and bodyweight. | Persuasion Enablement |
| 15.3 | ***Focus on past success*** | Advise to think about or list previous successes in performing the behaviour (or parts of it) | Advise the person to describe or list previous times when SB was replaced with non-SB. | Persuasion Enablement |
| 15.4 | ***Self-talk*** | Prompt positive self-talk (aloud or silently) before and during the behaviour | Prompt the person to tell themselves that all movement matters and / that or a movement break will be energising, will be worth it, is maintaining mobility, enhancing health. | Training  Enablement |
| 16.3 | ***Vicarious consequences*** | Prompt observation of the consequences (including rewards and punishments) for others when they perform the behaviour | Prompt person to observe a PwO in a movement break feeling pain free and happy;  Draw attention to the positive comments other PwO get when they reduce SB | Enablement |

**Table S9.** Intervention features and mode of delivery suggestions mapped to TDF and COM-B

| **Intervention suggestions Mapped to Theoretical Domains Framework and COM-B Model** | | | | |
| --- | --- | --- | --- | --- |
| **COM-B  component** | **TDF Domains** | **Intervention features** | **Mode of delivery** | **Style of delivery** |
| Physical  capability | Skills (physical) | improve ability, belief re ability coping skills pain, lack of ability, Pacing /boom bust alternatives, **Physical Activities suggested**  achievable steps, slower,  100 steps X 10 ad breaks, 2-10 x sit / stand, butt clenches, active gaming adapted Zumba, Yoga, Soft tennis, movements / exercise,  Balance exercises at home, Body realignment, gait retraining, chair exercise, yoga, pedals, step for push up, exercise for purpose, orienteering, family orienteering, exercises, simple program, graded, standing, chair, resistance, strength, weights, flexibility, at home, Just getting up, Just mobility.  walking, LPA, short walks, morning, evening, | adapted exercises at home;  virtual /online class;  exercises in an email or print (place on wall) Virtual walks  Walk media challenge active gaming exercise group / class for PwO, adult only,  movement at home, Online class, weekly |  |
| Psychological capability | Knowledge | **education** of PwO, HCP, GP, Providers care /intervention, public, school re complexity, factors influencing SB, occupational SB, re obesity (as disease, complexity, treatments, care, setpoint) re access to supports, to adapt exercises, re health consequences (SB, Obesity)  re health benefits (Non SB, LPA, comfort breaks daily mobility, empathetic) re **home movement**, staying mobile,  **Self-awareness**, re need for change | Webinars In a virtual online class PwO Awareness Ad campaign, flyers, magazines, social media, TV, Population level awareness, Media campaign target SB / movement; Slogan (advertising), social media (Instagram); | not didactic, progress not perfection, |
|  | Skills (cognitive and interpersonal) | Skills to change intention to action; planning for action, plan, Coping strategies / skills for internalised bias / Social stigma,  Coping skills, Emotional coping skills, not meeting goals, (non-achievement), internalised bias, Skill to restart, reset goal, re-engagement, Resiliency skills,  Self advocacy skills, | In a virtual online class PwO |  |
|  | Memory, attention and decision processes | Awareness Ad campaign,  Population level awareness,  prompts, vibration, feedback (some dislike)  Self-monitoring diary, | Awareness Ad campaign, flyers, magazines, social media, TV, Population level awareness, Media campaign target SB / movement; Slogan (advertising), social media (Instagram); prompts, vibration, feedback (some dislike)  Self-monitoring diary |  |
|  | Behavioural regulation | change intention into action, Measuring steps, self-monitoring, Monitoring by others, social support prompt,  motivation, planning for breaks, prioritise self, Self-monitoring diary  Lean mass feedback | reminders; prompts;  Monitoring; self -diary; pedometer steps;  Watch with feedback. Monitoring others; phone call from coach/ HCP.  Body composition scales, |  |
| Physical  opportunity | Environmental context  and resources | **Home,** treadmill, pedals, weights, walking DVD, Body composition scales, step for push-up Phone prompts, feedback, App, MapMyWalk app, Device watch (Apple apps), prompts, vibration, feedback  Fitbit, pedometer, steps, LPA, discreet prompts, electric bikes, Social media challenge walk x miles, | walking DVD  Phone prompts, App, MapMyWalk app, Device watch (Apps), prompts, vibration, feedback  Fitbit, pedometer, steps, LPA, discreet prompts,  Social media challenge (walk x miles), | **Policy** ; medication pain, obesity (Ozempic, semaglutide) SSRI, dexa scan, funded developed and provided by health service  commercial/ private funded, affordable intervention,  gyms, activity) |
| Social  opportunity | Social influences | bucket list group, Facebook group, women's shed, Branding targeted movement exercise class for PwO similar to senior classes, gentle movement,  professional led class, yoga/physio, movement Friends, selective buddies,  one on one Peer support, for activity (online / local) safe technique instruction social support for acceptance of 'failure', activity, problem solving, change of mindset,  encouragement, to remove barriers, to review goals Social support HCP, informed Coaches, psychology, mental health, WMC, Obesity supports,  Social support, monitoring by others, personal prompt if not engaged, encouragement Support and education during illness | bucket list group, Facebook group, women's shed, Branding targeted movement exercise class for PwO (similar to senior classes), gentle movement, professional led class, yoga/physio, movement Friends, selective buddies,  one on one Online class (movement), appropriate age, anonymity (optional), with PwO, consistent HCP, weekly, online peer video Peer support, for activity (online / local) encouragement (by phone -monitoring behaviour by others) walking group, partners |  |
| Reflective motivation | Social & professional  role and identity | Prioritise self; Self-love |  |  |
|  | Beliefs about capabilities | Physical ability |  |  |
|  | Optimism | realistic expectations (weight loss) |  |  |
|  | Intentions | stabilising intention (changing intention to action) |  |  |
|  | Goals | goal setting / resetting, achievable / realistic goals, coping skill not meeting goals, Goal 10000 steps, review goals, support to review, small achievable goals, frequently, |  |  |
|  | Beliefs about consequences | health consequences (SB, Obesity) obesity (as disease, complexity, treatments, care, set-point) health benefits (Non SB, LPA, comfort breaks daily mobility,  home movement. staying mobile, |  | empathetic |
| Automatic motivation | Reinforcement | changing social norms, non-shaming messages, skills for / break internalised bias, Stigma coping strategies for PwO stop weight bias in healthcare, Society  emotional reward, encouragement non-comparative, personal successes,  positive reinforcement, personal achievement, small victories reward for movement, small improvements,  socially rewarding, |  | non-shaming messages,  encouragement non-comparative |
|  | Emotion | acceptance / realism of 'failure'; lack of physical ability; Obesity; self as PwO; physical body;  confidence,  Emotion, want to move.  Emotional coping skills, (non-achievement), emotional reward,  encouragement to revise / restart, Enjoyment, Fun, ( activity, movement, enjoyment as decision ) reduce fear of gyms, resilience, psychological  Responsibility, (self, obesity,) self-love, self-motivation,  shared experiences, emotions, |  | acceptance and empathy  encouragement, (non-dictatorial, not instructions, e.g. have you tried…), |
